# Current Advancements in Transdermal Biosensing and Targeted Drug Delivery

**DOI:** 10.3390/s19051028

**Published:** 2019-02-28

**Authors:** Prem C. Pandey, Shubhangi Shukla, Shelby A. Skoog, Ryan D. Boehm, Roger J. Narayan

**Affiliations:** 1 Department of Chemistry, Indian Institute of Technology (BHU), Varanasi 221005, India; sbshukla1990@gmail.com; 2 Joint Department of Biomedical Engineering, University of North Carolina and North Carolina State University, Raleigh, NC 27695, USA; saskoog@gmail.com (S.A.S.); ryandboehm@gmail.com (R.D.B.)

**Keywords:** Iontophoresis, transdermal biosensing, drug delivery, electroporation, microfabrication, microfluidics, microneedle, luminescent sensors, fluorescent biosensors

## Abstract

In this manuscript, recent advancements in the area of minimally-invasive transdermal biosensing and drug delivery are reviewed. The administration of therapeutic entities through the skin is complicated by the stratum corneum layer, which serves as a barrier to entry and retards bioavailability. A variety of strategies have been adopted for the enhancement of transdermal permeation for drug delivery and biosensing of various substances. Physical techniques such as iontophoresis, reverse iontophoresis, electroporation, and microneedles offer (a) electrical amplification for transdermal sensing of biomolecules and (b) transport of amphiphilic drug molecules to the targeted site in a minimally invasive manner. Iontophoretic delivery involves the application of low currents to the skin as well as the migration of polarized and neutral molecules across it. Transdermal biosensing via microneedles has emerged as a novel approach to replace hypodermic needles. In addition, microneedles have facilitated minimally invasive detection of analytes in body fluids. This review considers recent innovations in the structure and performance of transdermal systems.

## 1. Introduction

Transdermal drug administration and biosensing has become more widely accepted in recent years. The skin typically exhibits a triple-layered structure, with outermost epidermis, middle dermis, and the innermost subcutis. The dermis is fully vascularized and possesses sweat glands, hair follicles, nerve endings, and lymph vessels. This layer of tissue facilitates the local absorption of drugs. The intercellular lipid bilayer of the stratum corneum, the uppermost layer, constitutes the rate-limiting layer for the migration of hydrophilic drug molecules across it [1,2,3]. Several methods have been investigated to increase the permeation rate of drugs temporarily and locally, including chemical and electrical mechanisms [4,5]. Chemical agents elevate the rate of permeability of drug molecules by improving the diffusion rate through the stratum corneum [6]. Electrical magnification of biosensing and drug transport involve two methods: electroporation and iontophoresis [4,5,7,8]. Physicochemical techniques like microneedles [9,10], ultrasound [11,12], and nanomaterials act as driving forces in promoting the transdermal migration of molecules. This review summarizes advance technologies that have been developed for transdermal biosensing and drug delivery. 

## 2. Electrical Amplification

Several strategies have been employed to facilitate the drug delivery and sampling of body fluids for transdermal biosensing applications. These approaches are considered in the following subsections.

### 2.1. Iontophoresis

**Figure 1:**
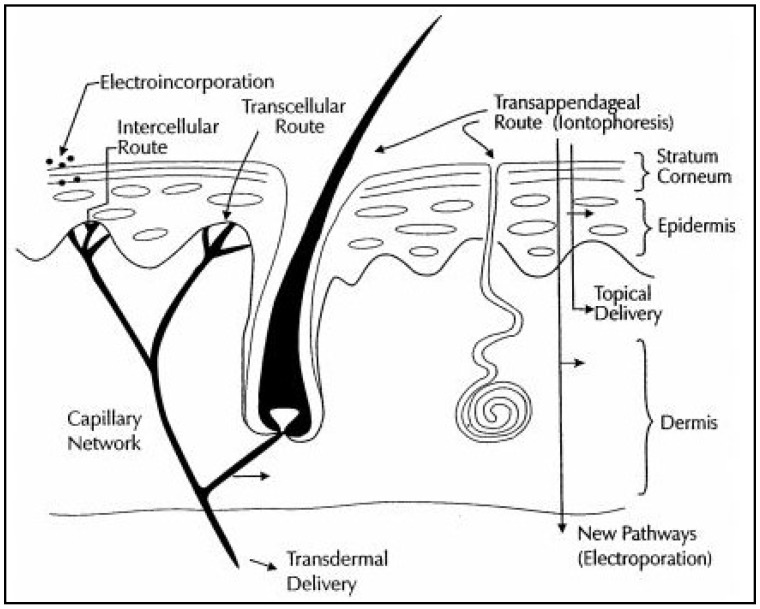
Schematic illustration showing the pathways of topical and transdermal delivery, including electrically assisted delivery by iontophoresis, electroporation, and electroincorporation [7].

Iontophoresis utilizes a small voltage (usually <10 V) and a small current density (<0.5 mA/cm^2^) to drive charged drug molecules through the skin, utilizing an electrode with the same charge as the molecule that is being delivered [4,5,7]. It increases permeation of ionic drugs, provides programmable drug delivery, improves patient compliance with electronic reminders, allows patient-specific delivery controlled by current adjustments. A few devices using this approach have been commercially translated. This process is believed to drive drugs through preexisting pathways; however, it should be noted that the low voltage could result in new pores or pathways. Another similar technique is electroporation, which uses a high voltage pulse (>100 V) applied for a short time period (microseconds-milliseconds) to create pores or channels in the lipid bilayer membranes in a reversible manner. This approach has been utilized to transfect cells and it can also be used to deliver drugs across the skin. It takes approximately 1 V per membrane to cause pore formation. This technique can allow for delivery of large molecules, DNA, oligonucleotides, and other relevant molecules. Combining this technique with the electroporation process may improve transdermal drug delivery as electroporation opens up channels [8] and iontophoresis drives the molecules through them (Figure 1).

### 2.2. Iontophoresis in Combination with Electroporation

#### 2.2.1. Particulate Delivery

This approach involves the encapsulation of drug molecules in vesicles or particles; a current or a pressure gradient then promotes movement of the encapsulated drug through electrically created pores in the skin. The movement of drugs is believed to be the result of a combination of several mechanisms, governed by the Nernst-Planck equation and Fick’s first law of diffusion. Variations/combinations of these equations have been proposed which explained the combined effects of pore formation, passive diffusion, and electrophoresis. A variety of in vitro tests have been conducted to study transport, including: (a) horizontal or vertical types (e.g., Franz diffusion cells) of diffusion cells and (b) synthetic membranes (e.g., Nucleopore^®^) to mimic skin; due to the high variability of skin tissue, it is difficult to find membrane materials that possess all of the characteristics of skin (e.g., a combination of hydrophobicity and conductivity).

The use of chemical enhancers to improve transdermal delivery has been shown to provide synergy with electrotransport of drugs (e.g., iontophoretic delivery of LHRH; use of heparin with electroporation may keep pores open longer). It should be noted that a residual decrease in skin resistance (i.e., increased skin permeability) has been observed in excised skin. Several studies have been conducted on iontophoresis and electroporation using excised skin and porcine models, which generally indicated the low levels of side effects (e.g., erythema) and a good recovery of skin resistance [4,5,7,8]. The problem of drug load dumping observed from a short circuit can be effectively controlled with improved circuitry design.

#### 2.2.2. Clinical Dermatological Applications

Iontophoresis has been used in a variety of clinical applications. Shimizu et al. noted that the iontophoresis using direct current causes burns and pain [13]. They used iontophoresis with alternating current in conjunction with anticholinergic drug delivery to reduce perspiration among palmoplantar hyperhydrosis patients. It was noted that alternating current iontophoresis along with anticholinergic drug delivery caused a more rapid reduction in perspiration than alternating current iontophoresis alone.

#### 2.2.3. Protein Delivery

Iontophoresis may aid in the pulsed delivery of large peptides, which would otherwise face difficulties in transdermal diffusion [14,15] (e.g., thyrotropin-releasing hormone, vasopressin, calcitonin, and luteinizing hormone-releasing hormone (LHRH)). Electroporation has also been investigated to improve transdermal LHRH delivery. Insulin has been previously delivered under the combined action of electroporation and iontophoresis processes. Insulin monomers should be able to penetrate the skin; however, the high molecular weight of synthetic insulin (hexamer) is not compatible with passive transport. Iontophoretic delivery may result in the formation of depots containing the drug in the skin. The combination of electroporation and iontophoresis may allow for controlled transdermal delivery with skin loading and release.

## 3. Applications of Iontophoresis

### 3.1. Iontophoresis in Drug Delivery

Iontophoresis is known for enhancing drug permeation through the scarred epidermis. Compared with invasive techniques such as laser therapy, radiotherapy, cryotherapy, pressure therapy, and intralesional injections, iontophoresis is less expensive and free of discomfort. Every dermal injury results in scarring, which is a common consequence of the tissue repair process. Extra deposition of collagen tissue during wound healing creates a tough epidermis at the injury site, which restricts the passive transport of drug through it. Iontophoresis supports drug delivery across the scar epidermis [16] and also through the skin, which lacks peripheral circulation pathways. For instance, dead skin cells lack connections with the systemic circulation; iontophoresis assists with drug transport beyond the dead skin layer. In earlier reports, it was established that iontophoresis facilitated the movement of terbinafine across the nail fold epidermis at a much higher rate (150×) than unassisted passage of the drug. In another case, iontophoretic delivery across folded porcine epidermis in which the fending off passages were essentially blocked was thought to occur via imitating shunts. Iontophoresis reportedly enhanced the transport flux of sodium fluorescein (a marker to track the transport of the drug) across the scar skin epidermis to 51.9 ± 8.82 ng/(cm^2^ h), a rate 46-fold higher than that associated with passive permeation.

As expected, iontophoresis also enhanced the transport flux of sodium fluorescein across the normal skin epidermis by approximately 200-fold over normal skin epidermis; a flux of 601.24 ± 62.38 ng/(cm^2^ h) was noted. The iontophoretic motion of sodium fluorescein beyond the scar epidermis was enhanced several fold as compared with the normal epidermis; epidermal drug retention was also increased by iontophoresis. These results indicate that iontophoresis is an appropriate approach for the delivery of therapeutic agents across scarred skin for scar treatment.

In another report, reverse iontophoresis was studied as a sensing mechanism for transdermal drug monitoring, utilizing the “internal standard” calibration approach [17]. Various concentrations of sodium valproate (21 µM–1 mM) and a fixed concentration of glutamic acid (60 µM) were introduced into the subdermal area of full-thickness excised porcine ear skin. The recovered valproate ions were compared to the glutamate ions (serving as the standard) as a means of determining the subdermal valproate concentration. 

**Figure 2:**
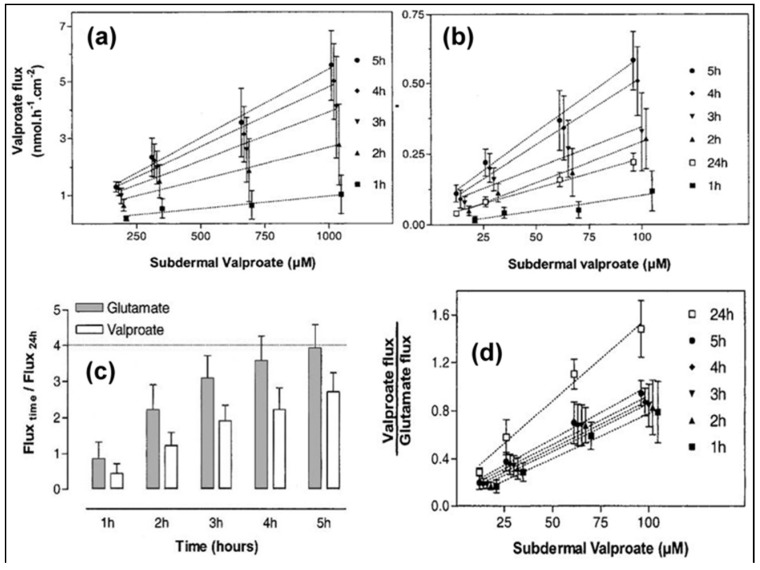
Reverse iontophoretic extraction flux rates of valproate as a function of time and subdermal concentration (**a**) in the range 209–1050 μM, and (**b**) in the range 21–104.5 μM. Reverse iontophoretic extraction flux rates of valproate and glutamate as a function of time relative to their values at 24 h. (**c**) Ratio of the reverse iontophoretic extraction flux rates of valproate and glutamate as a function of time and subdermal valproate concentration (range 21–104.5 μM). (**d**) Each data point represents the mean ± standard deviation (n = 6) [17].

The results, shown in Figure 2, indicated that the internal standard method could be used successfully to determine the valproate concentration; the valproate/glutamate ratio was confirmed to be dependent on the subdermal valproate concentration while the glutamate level remained constant. 

**Figure 3:**
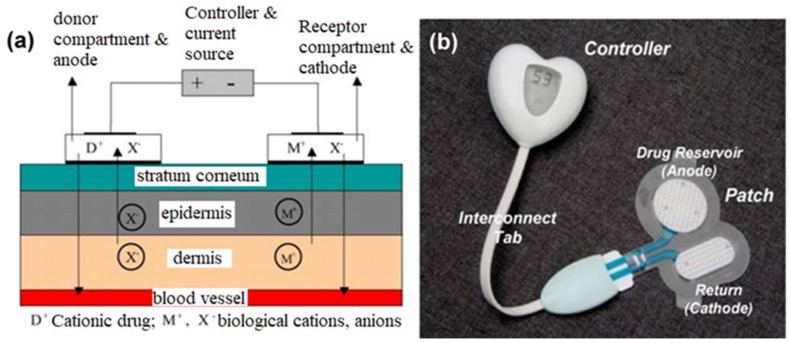
(**a**) An iontophoretic drug delivery system comprising donor and receptor compartments along with a current source and controller. D^+^: cationic drug; M^+^: biological cations; X^−^: biological anions. (**b**) Vyteris Inc. LidoSite^TM^ topical system [18].

Subramony et al. described transdermal drug delivery using the iontophoresis process; they considered the delivery of lidocaine for anesthesia and fentanyl for post-operative pain [18]. The general process of iontophoretic drug delivery involved an electrode patch with positively charged ions driven transdermally by the anode (accepting negative ions such as chloride from the body); the cathodic counter electrode accepts the positive biological ions (e.g., sodium and potassium ions) while displacing negative ions from its reservoir (Figure 3). This drug delivery function or ionic flux is based upon a derivation of Faraday’s law:

*J_d_* = *t_d_IM_d_*/*Z_d_F*,

where *J_d_* is the ionic flux, *t_d_* is the drug transport number, *I* is the current density, *M_d_* is the molecular weight of the drug ion, *Z_d_* is the charge of the drug ion, and *F* is Faraday’s constant.

### 3.2. Iontophoresis in Transdermal Biosensing

#### 3.2.1. Transdermal Glucose Monitoring

**Figure 4:**
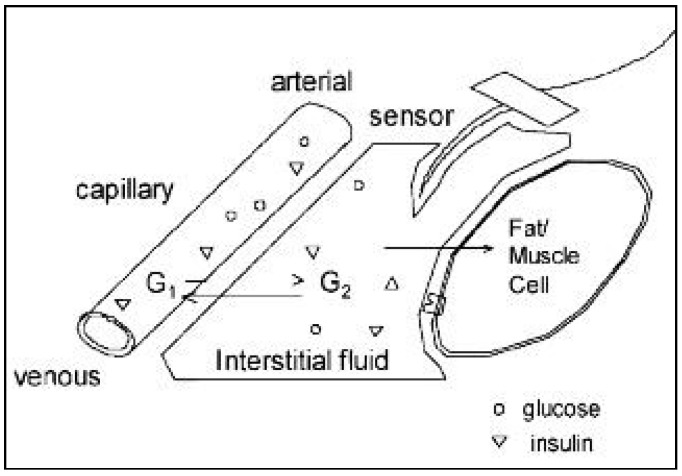
Glucose electrode inserted in subcutaneous tissue. Glucose diffuses from the intravasal compartment (G_1_) into interstitial compartment (G_2_0; it is then taken up by cells if insulin is present [19].

Use of an iontophoretic approach to extract interstitial fluid for glucose sampling has been considered; it is important to correlate the blood glucose levels with the glucose levels in the interstitial fluid. Approaches include reverse iontophoresis, microporation of the skin, patch delivery of permeation enhancers, ultrasound to increase transdermal flux, and fluorescence tagging of glucose (Figure 4). Reverse iontophoresis, which uses low levels of current to transport glucose from interstitial fluid to the sensor interface, is the approach utilized by the GlucoWatch Biographer (GlucoWatch; Cygnus, Redwood City, CA, USA); this approach allows for acceptable measurements of glucose [19]. Local skin irritation has been noted with this approach; in addition, the device cannot be worn during heavy perspiration. 

SpectRX (Norcross, GA, USA) described a technique that creates micropores in the skin by laser burning, which facilitates the transdermal transit of ions for several days. TCPI (Fort Lauderdale, FL, USA) described the use of a patch with a permeation enhancer, which allows for glucose readings to be taken with a glucose meter in the affected area. 

**Figure 5:**
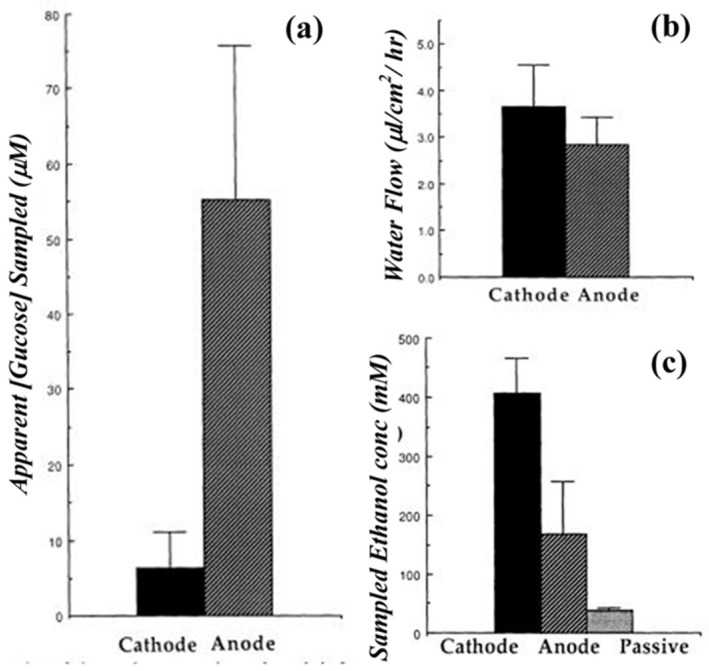
(**a**) Apparent extraction of glucose by reverse iontophoresis in 2 h. Reverse iontophoretic extraction of (**b**) titrated water and (**c**) ^14^C-labeledethanol in 2 h [20].

In another report, in vitro reverse iontophoresis was investigated as a mechanism for glucose monitoring in full-thickness skin of hairless mice using unlabeled and radiolabeled (^14^C-labeled) glucose solutions [20]. The experiments utilized both platinum/glucose oxidase (Pt-GOD) and modified copper electrodes for the delivery of current (0.36 mA/cm^2^) over the course of two hours. The Pt-GOD electrode was glucose specific, and the modified copper electrode was able to oxidize a variety of organic species that contain hydroxyl groups. The results of the study indicated glucose was able to be electroactively transported through the skin at concentrations proportional to the solution bath to which the dermis was exposed. It was noted that a higher degree of radiolabeled analyte was located at the anode than expected; the authors attributed this observation to metabolic breakdown of glucose into negatively charged metabolites (e.g., lactate and pyruvate) that would be drawn to the anodes of both electrodes and the lack of glucose specificity by the modified copper electrode. A correction for this unexpected signal on the Pt-GOD electrode was accomplished by incorporating ascorbic acid oxidase into the process to remove ascorbic acid that was drawn to the anode (Figure 5). The study demonstrated the potential for this approach as a non-invasive transdermal glucose sensing modality; however, additional research is needed to determine its feasibility in vivo.

A transdermal biosensing approach involving temporary tattoo-based epidermal diagnostic device combining reverse iontophoretic extraction of interstitial glucose and enzyme-based amperometric biosensor has been recently developed [21]. This reverse iontophoretic biosensing system involves a specific type of electrode rather than the typical three-electrode electrochemical biosensing approach. The system involves anodic and cathodic parts, each of which consists of an Ag/AgCl reference electrode with reverse iontophoretic working and counter electrodes.

**Figure 6:**
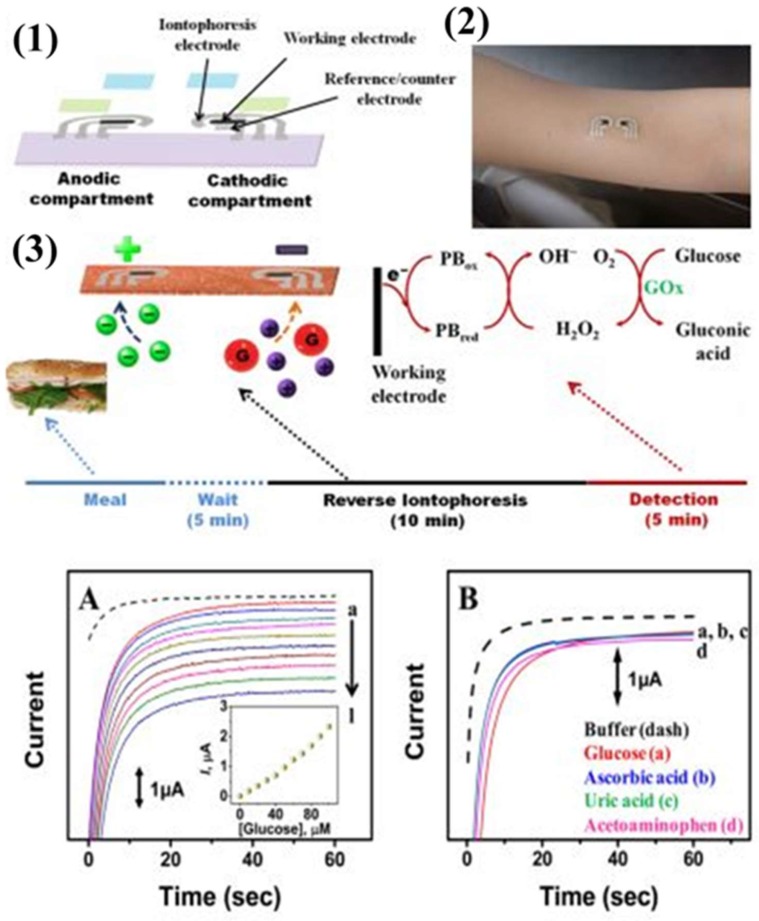
Tattoo-based platform for noninvasive glucose sensing. (**1**) Schematic of the printable iontophoretic-sensing system displaying the tattoo-based paper (purple), Ag/AgCl electrodes (silver), Prussian Blue electrodes (black), transparent insulating layer (green), and hydrogel layer (blue). (**2**) Photograph of a glucose iontophoretic sensing tattoo device applied to a human subject. (**3**) Schematic of the time frame of a typical on-body study and the different processes involved in each phase. (**A**) Chronoamperometric response of the tattoo-based glucose sensor to increasing glucose concentrations from 0 μM (dashed) to 100 μM (plot “l”) in buffer in 10 μM increments. (**B**) Interference study in the presence of 50 μM glucose (plot “a”), followed by subsequent 10 μM additions of ascorbic acid (plot “b”), uric acid (plot “c”), and acetaminophen (plot “d”). Potential step to −0.1 V (vs. Ag/AgCl). Medium was phosphate-buffer with 133 mM NaCl (pH 7) [21].

In this reverse iontophoretic process, extraction of interstitial fluid containing glucose occurs at the cathode. As such, it is modified with the glucose oxidase enzyme for the selective detection of glucose in the presence of uric acid, ascorbic acid, or acetaminophen. Issues like skin irritation and biocompatibility were overcome by applying a uniform coating of agarose gel, which also maintains better contact between the tattoo and the skin. This sensor responded linearly over the concentration range of 0–100 M, with a limit of detection of 3 M during in vitro studies (Figure 6). On body use of this reverse iontophoretic-biosensing platform could monitor changes in blood glucose levels without entering the skin.

Alvarez-Lorenzo and coworkers discussed the use of molecularly imprinted polymers (MIPs), mainly as drug delivery systems (DDSs); they also mentioned potential applications of MIPs in sensor devices. In feedback-regulated drug delivery systems, the MIPs release drugs based on recognition of a molecule of interest; this approach could also be used in transdermal sensing systems [22]. The highest profile example of this approach is the use of a MIP sensor for glucose sensing in a DDS for insulin.

**Figure 7:**
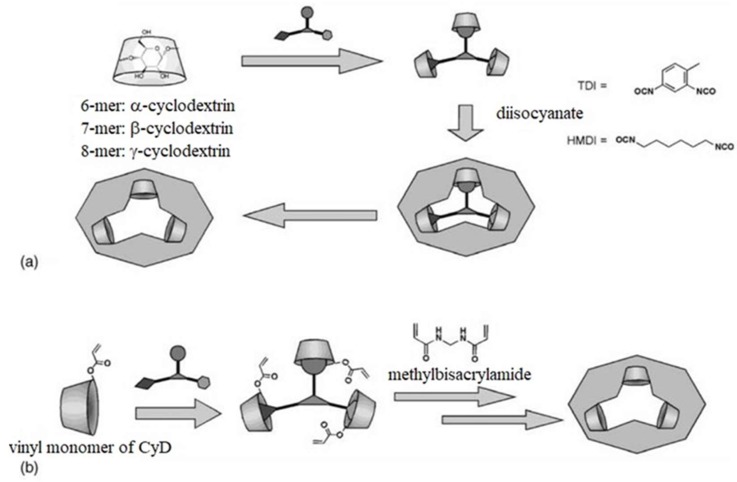
Molecular imprinting of cyclodextrins as receptors of nanometer-scaled templates. (**a**) The cyclodextrin is cross-linked by diisocyanate, in dimethylsulfoxide, in the presence of the template. (**b**) The vinyl monomer of cyclodextrin is copolymerized with methylenebisacrylamide, in water, in the presence of the template [22].

These devices have been evaluated for determining blood glucose levels; a number of studies have been conducted to correlate glucose concentrations with pH changes based on (a) metal coordination due to interactions of functional monomers and cross-linking agents (e.g., [4-(*N*-vinylbenzyl)diethylenetriamine) copper(II)] diformate and [(diethylenetriamine) copper(II)] dinitrate), (b) non-polar and polar cross-linking agents for carbohydrate binding discrimination, and (c) insulin releasing gels that react to glucose concentration changes by glucose hydroxyl group interactions with 3-acrylamidophenylboronic acid monomers (Figure 7). In addition, glucose traps have been studied; for example, a non-covalent network created by ionic associations of poly(allylamine hydrochloride), glucose phosphate monosodium salt, and various cross-linking agents has been evaluated. While these approaches have been developed for capturing ingested glucose, limiting glucose uptake, and preventing spikes in blood glucose values, they may also be useful in sensing systems.

Liu et al. investigated transdermal glucose monitoring by reverse iontophoresis using a 3-electrode cell (with a gold working electrode as well as Ag/AgCl reference and counter electrodes) on a polycarbonate sheet; a hydrogel membrane served as a reaction area [23]. The working electrode was functionalized with an Os-complex mediator layer (horseradish peroxidase (HRP) and poly(ethylene glycol) diglycidyl ether (PEG-DGE)). The hydrogel layer was composed of poly(ethylene oxide) (PEO) (4% *w*/*v*), bovine serum albumin (3% *w*/*v*), glucose oxidase (2 U/µL), and glutaraldehyde (0.5% *w*/*v*).

Amperometric characterization of the sensor indicated high sensitivity (14.24 nA uM^−1^cm^−2^) and a good correlation coefficient compared to the calibration standard (r = 0.99). Additionally, since the characterization was completed at −0.1 V (versus Ag/AgCl), interference from electroactive analytes (e.g., uric acid) was avoided; the GlucoWatch^®^, which operates at 0.42 V compared to its reference, cannot avoid this complication. Furthermore, the reverse iontophoretic experiments on nude mouse skin showed the ability to detect glucose in the 0–18 mM range, with a linear relationship (r^2^ = 0.99, which is better than the r^2^ = 0.98 of the GlucoWatch^®^), and the ability to switch polarity of the electrodes at 20 min intervals. It is hypothesized that reversing polarity periodically during the sensing process can help to eliminate irritation at the skin-electrode interface.

**Figure 8:**
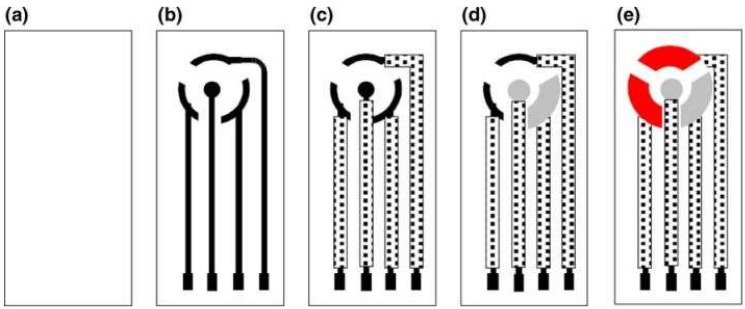
Construction steps for the planar configuration of the screen-printed amperometric transducer. (**a**) PETE support material; (**b**) printing of conducting silver basal track; (**c**) printing of insulation layer; (**d**) printing of Ag/AgCl pads, the central circular pad is the iontophoresis electrode for reverse iontophoresis while the other pad is the reference electrode; (**e**) printing of graphite pads, which serve as the working and counter electrodes [24].

In another study, a reverse-iontophoretic glucose sensor for diabetic monitoring of glucose levels was considered [24]. It was noted that the only commercially available reverse iontophoretic glucose sensor is the GlucoWatch^®^ biographer; there are reports of skin irritation that was caused by the device. In addition, there are inconsistencies in the ability of the device to diagnose hypoglycemia and hyperglycemia in patients (Figure 8). 

The authors hypothesized that the cause of patient skin irritation involved the use of glucose oxidase on the electrode without a redox mediator, effectively trapping hydrogen peroxide against the skin; the peroxide is also harmful to the glucose oxidase and may limit the effectiveness of the sensor. To combat these issues, the authors sought to create an amperometric device with ferrocene as a redox mediator for the glucose oxidase. The device was created by a series of screen-printing steps of Ag, Ag/AgCl, and graphite to form the electrodes on poly(ethylene terephthalate) sheets; one of the graphite electrodes was subsequently functionalized by successively drop-casting ferrocene and glucose oxidase solutions (Figure 8). The amperometric results of the device showed a linear range for glucose sensing (0-4 mM) and a response time of 10 s. When tested on a trans-membrane with reverse iontophoresis, the device was able to detect glucose levels with a good linear relationship (r^2^ = 0.99) compared to the known concentrations (3, 5, and 15 mM); these concentrations mimicked hypoglycemic, normal, and hyperglycemic conditions. The authors concluded that the ferrocene-mediated glucose sensor may be useful for applications in reverse iontophoretic transdermal sensing; a fast response time, reproducibility, and good sensitivity were demonstrated. 

#### 3.2.2. Transdermal Sampling of Urea and Homocysteine

Ching et al. investigated non-invasive transdermal extraction of homocysteine and urea simultaneously by reverse iontophoresis [25]. Earlier reports have described an enhancement in the extraction of urea using the reverse iontophoresis approach. An in vitro model consisting of three layers that resemble the triple-layered pattern of skin (epidermis, dermis, and subcutis), was used to detect urea and homocysteine. The diffusion cell imitated the function of the dermis and subcutis layers; porcine skin with a thickness of 240 μm replicated the epidermis. Electromigration of urea, which is an ionic molecule, and electro-osmosis of homocysteine, which is an uncharged and hydrophilic molecule, facilitate transport across the layers. 

Diffusion in combination with reverse iontophoresis amplified the extraction of homocysteine; there was much higher extraction of homocysteine for direct current (dc) and four symmetrical biphasic direct currents (SBdc) than for samples that relied solely on diffusion. Further, it was observed that the transfer of homocysteine was lower at the dc-anode and dc-cathode than for the four SBdc approach. This result is attributed to polarization of the porcine skin associated with the usage of direct current, which retarded the migration of homocysteine (neutral molecule) during reverse iontophoretic operation. Furthermore, similar behavior was observed in the case of the extraction of urea; reverse iontophoretic operation facilitated greater levels of extraction than the diffusion process. Urea is negatively charged; as such, its extraction is more reinforced at the dc anode than at the dc cathode. Urea also followed phase-duration dependent extraction in case of SBdc. The extraction of urea was found to be optimum when 60 s PDSBdc was employed. The reverse iontophoretic operation served as an appropriate approach for transdermal extraction of both substances.

## 4. Microfabrication Technique for Transdermal Sensing and Drug Delivery

**Figure 9:**
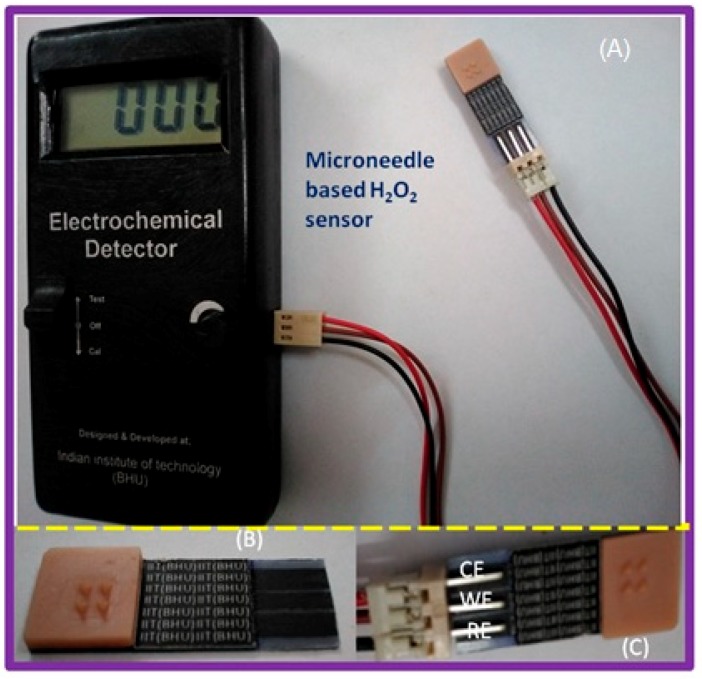
Construction of microneedle assembled screen-printed electrode (SPE)-based amperometric sensor for hydrogen peroxide. (**A**) Electrode assembly connected to a dedicated electrochemical detector; (**B**) Microneedle assembled SPE in three electrode configuration; (**C**) Electrode connector fixed to SPE: WE = working electrode, RE = reference electrode and CE = counter electrode) [26].

**Figure 10:**
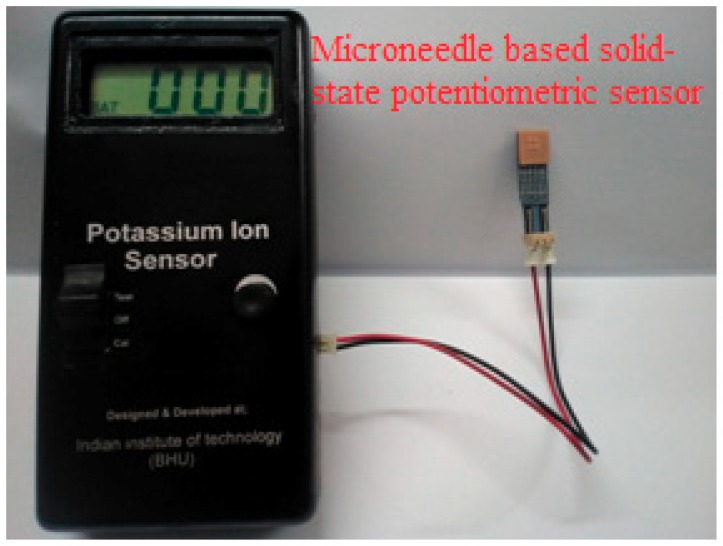
Construction of a microneedle assembly-based screen-printed electrode (SPE)-based potentiometric potassium ion sensor [27].

Microfabrication techniques have been used to create micro- and nano-scale features; for example, the techniques may be used to: (a) impart desirable surface properties, (b) create circuitry for electronic components, and (c) facilitate high-throughput and large-scale production of devices with medical relevance. These techniques can aid in the creation of micro-scale devices and biosensors for transdermal sensor applications [26,27,28]. For instance, microfabrication techniques can be employed to fabricate microneedle devices for penetration of the skin into the epidermis or dermis. Previous studies have created arrays with up to thousands of microneedles (noted diameters of 10–100 µm) out of polymers and silicon for delivery of therapeutic agents, vaccines, or DNA. Furthermore, micromolding the master structures of the microneedles can enable processing of microneedles out of biodegradable materials (e.g., carboxymethylcellulose, amylopectin, polylactic acid, polyglycolic acid, and poly(lactic-co-glycolic) acid). Furthermore, these microfabrication techniques can be used in the development of biosensors (e.g., electrochemical or mechanical loading sensors). We have recently demonstrated a screen- printed electrode in the three-electrode configuration, including working (WE), reference (RE) and counter (CE) electrodes, as shown in Figure 9. The working electrode was modified with a catalytic layer for detection of hydrogen peroxide either generated at the electrode surface or in the sample. The active catalytic layer may include enzymes, palladium nanoparticles with controlled nanogeometries, or quantum dots [26]. The electrodes were printed over a flexible material, which can be integrated with a hollow microneedle assembly for transdermal sensing [26]. Similarly, a solid-state ion sensor in a two-electrode configuration assembled with a hollow microneedle was designed for transdermal sensing of electrolyte (shown in Figure 10) [27]. The all solid-state microneedle assembled potentiometric sensor was comprised of a PVC matrix membrane ion sensing layer over a non-specific ion carrier impregnated membrane made from a suspension of siloxane-polyindole-gold nanoparticles [27] to maintain a constant dipolar potential during potentiometric transdermal sensing. The reference electrode was an Ag/AgCl electrode covered with a *p*-toluenesulfonate impregated polyndole-gold nanoparticle-organically modified silicate membrane [27], which was combined with the microneedle-assembled electrode to yield a solid-state ion sensor.

Glucose oxidase is used in this manner in glucose meters for diabetic patients; microfabrication techniques can be utilized to (a) pattern the interfaces of these types of biosensors with the biological environment and (b) create the circuitry needed to transmit electronic signals produced by the enzymatic interactions. However, currently these devices have been investigated mainly for implantation (e.g., an implant in the rat peritoneal cavity for ethanol detection or a glucose sensitive sensor that releases antibiotics when glucose produced by bacteria at an orthopedic implant site changes the viscosity of the solution surrounding a microcantilever) and lab-on-a-chip applications [27,28].

## 5. Nanotechnology in Transdermal Biosensing

The review by Cintenza on the biomedical applications of quantum dots (QDs) touches on a few areas that are relevant to transdermal sensing [29]. Quantum dots are semiconductor nanocrystals in the size range of 0.2 to 100 nm (crystals in the size range of 2 to 10 nm are typically used). Due to the small size of these nanoparticles, they exhibit discrete energy levels with the behavior of the electrons and holes in their orbitals governed by quantum mechanics; as a result, small changes in their size (which can be controlled by fabrication techniques) cause changes in their fluorescence properties [30,31,32]. These nanostructures have tunable fluorescence properties by controlling their size, material composition, and fabrication temperature; consequently, they have the ability to outperform organic fluorophores due to their strong signal, tunable emission wavelengths, narrow emission profiles, and potential for use with only one light source for excitation for a variety of emission signals. While these nanocrystals have gained attention for a variety of biomedical applications (e.g., in vivo imaging, drug delivery systems), they also have applications in sensing technology [31]. The excellent optical properties of QDs have allowed them to function in various FRET assays for the detection of biologically relevant molecules (e.g., nucleic acids, proteins, and antibodies). 

**Figure 11:**
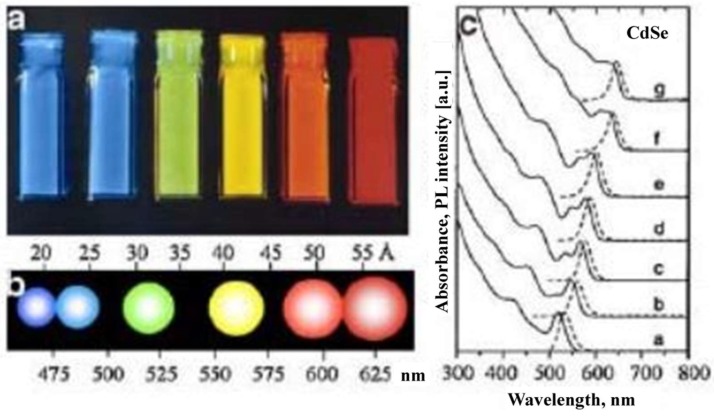
Size-dependent absorption and emission spectra of CdSe semiconductor nanocrystals with various sizes. (**a**) Size-dependent luminescence color and (**b**) schematic presentation of size, color, and emission wavelength of CdSe–ZnS QDs. (**c**) Absorption (solid lines) and emission (broken lines) spectra of CdSe QDs with various sizes [33].

The FRET characteristics of the QDs have led to the development of sensors for the detection of specific nucleic acid hybridization reactions (e.g., green-emitting CdSe/ZnS QD donors coupled with Cy3 and rhodamine RhR acceptor dyes) (Figure 11), detection of glucose levels in human serum (e.g., conjugated CdTe-conclavin A QD donors and gold nanoparticles with thiolated b-cyclodextrin modification as the acceptors), and detection of metabolic protease activities (e.g., CdSe/ZnS with a rhodamine-labeled tetrapeptide RGDC coating as the acceptor molecule).

## 6. Microneedles Technique in Transdermal Sensing and Drug Delivery

### 6.1. Polymer-Based Microneedles for Drug Delivery

Frisk et al. described the fabrication and testing of a microneedle array patch device (1 cm^2^) for transdermal drug delivery, featuring 200 µm tall microneedles that contained out-of-plane hollow bores, using a novel deep reactive-ion etching (DRIE) process [34]. The array was coupled with a heat-activated/printed circuit board-controlled (PCB) drug delivery fluid actuator made of silicone rubber, which expelled the drug load from a reservoir through the microneedles when heated. This concept was tested with Fast Green dye on the hands of human subjects and radio-labeled inulin in an in vivo rat model. The results of this study indicated that the fluid delivery rate in each case could be controlled by the input power (150–450 mW). A rate of 1 µL/min was applied in the human subjects, allowing for successful delivery of the Fast Green into the tissue; however, it was noted that this flow rate was too high for tissue absorption of all of the liquid. Residual dye remained on the skin surface after patch removal. In the rat study, a rate of 1.8 µL/h was utilized, with no observed inulin residue on the skin following patch removal. Sampling of the rat urine following fluid delivery indicated successful delivery of the radio-labeled ^3^H-inulin; sampling was conducted at 20-min intervals, with steadily increasing levels of the inulin observed. This result indicated that the drug was being delivered and absorbed into the circulatory system of the rats.

Targeted drug release using the microneedle technology has evolved recently as a second generation of minimally invasive means of transdermal drug delivery. Chen et al. reviewed NIR laser irradiation-triggered release of a drug from the microneedles at a directed site [35,36,37,38]. When the microneedles were irradiated with NIR light, the mobility of polymeric chains in the microneedles enabled drug release; the release was terminated when laser light was removed. The microneedles offer an effective means for drug administration in the body; the system is repeatedly switchable, which enables release of a consistent dosage of the drug. 

**Figure 12:**
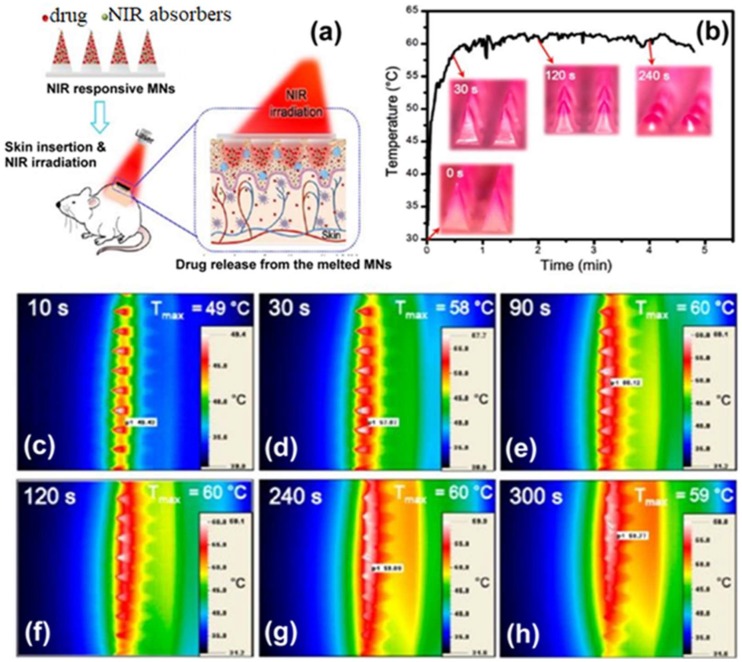
(**a**) Schematic illustrations of on-demand transdermal drug delivery using near-infrared (NIR) light-responsive microneedles (MNs), composed of polycaprolactone and NIR absorbers as well as silica-coated lanthanum hexaboride (LaB_6_-SiO_2_) nanostructures. (**b**) Temperature changes of R6G-loaded microneedles after continuous exposure to 808 nm NIR laser light at an output power of 7 W cm^−2^. (**c**–**h**) Infrared thermal images of microneedles after continuous irradiation for 10, 30, 90, 120, 240, and 300 s. The insets in (**b**) show the light-triggered melting behavior of microneedles [37].

In another study, solid and robust R6G-loaded microneedles were replicated from a master structure [37]. The quantity of drug liberated by the formulated microneedles could be altered by changing the number of on/off laser cycles. In vitro drug insertion was studied using porcine cadaver skin tissues and R6G as model drug; an application force of 10 N/patch was used in this study (Figure 12). Balanced forces facilitated the movement of microneedles during the puncture by subsuming the skin resistance. In general, a larger insertion force was associated with a greater insertion depth and a higher level of drug permeation. In vivo applicability of NIR absorptive microneedles were evaluated by loading the devices with an anti-cancer drug known as doxorubicin (DOX) into rat skin with four on/off laser cycles. The NIR-light responsive microneedles are a promising transdermal drug delivery system as they offer controlled and on-demand drug release.

Ashraf et al. described a method for designing and fabricating a transdermal drug delivery system consisting of out-of-plane hollow silicon microneedles, which are coupled to a valve-less piezoelectric actuated micropump [39]. They conducted theoretical analyses to understand the mechanical function of the microneedles, the piezoelectric mechanism of the pump, and computational fluid dynamics; a multifield computational examination of the entire working device was also performed. The microneedles were fabricated using a combination of deep reactive ion etching (DRIE), wet etching, and gas etching of masked silicon wafers; microneedles with an inner diameter of 60 µm, an outer diameter of 150 µm, a center-to-center spacing of 1000 µm, and high aspect ratio tips were created in this manner. The theoretical and numerical analyses of the system indicated that skin penetration of these devices could occur at 3.18 MPa; under these conditions, a maximum stress on the 3.26 MPa was observed with minimal deflection of the luminal structure (these values are below the yield strength of the material). When coupling the piezoelectric micropump to the system, the theoretical maximum pumping rate was determined to be 83.99 µL/min at 250 Hz and 100 V; it was also noted that this value corresponds to pulsatile flow, which is appropriate for pharmacologic treatment of some cardiovascular disorders (e.g., hypertension).

### 6.2. Microneedles for Chemical and Electrochemical Biosensing

**Figure 13:**
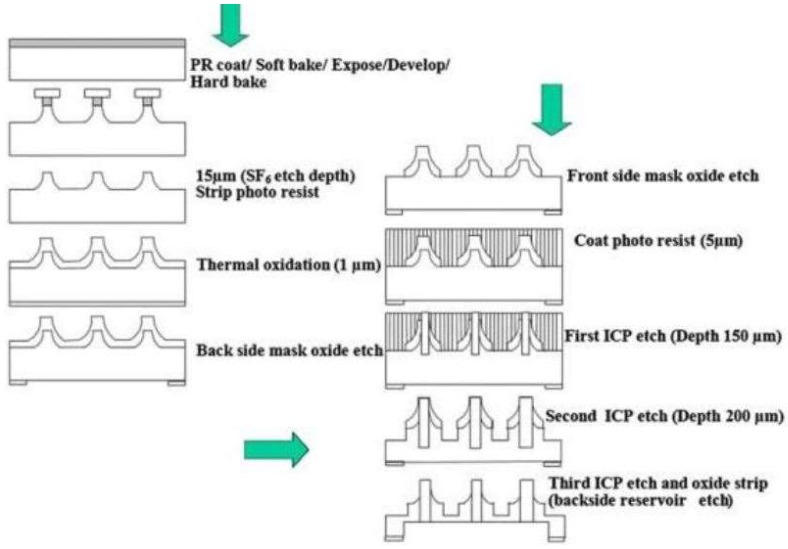
Fabrication process of hollow silicon out-of-plane microneedles [41].

Goud et al. fabricated an electrochemical microneedle-based biosensor and characterized the glucose sensor functionality of the minimally-invasive transdermal device [40]. The device contained an amperometric biosensor, a working electrode of carbon nanotubes/platinized glassy carbon within a sol-gel matrix of zirconia and Nafion; the electrode was functionalized with glucose oxidase. The electrodes were housed within a microfluidic chamber made of SU8 photoresist and sealed with polydimethylsiloxane (PDMS). Conceptually, the analytes were drawn to this microfluidic electrode chamber through organically modified silicates microneedles that had been fabricated by two-photon polymerization onto the external surface of the sensor unit (Figure 13). The microneedles exhibited base diameters of 100 µm and heights of a few hundred micrometers. Cyclic voltammetric and amperometric examinations of the sensor electrodes in glucose solutions (0–20 mM) showed nearly instantaneous responses times; the measured current values ranged from 1.0–2.5 µA as the glucose concentration in solution changed from 5–20 mM. This study demonstrated the potential to use an integrated biosensor device with microneedle arrays for transdermal sensing of glucose levels.

**Figure 14:**
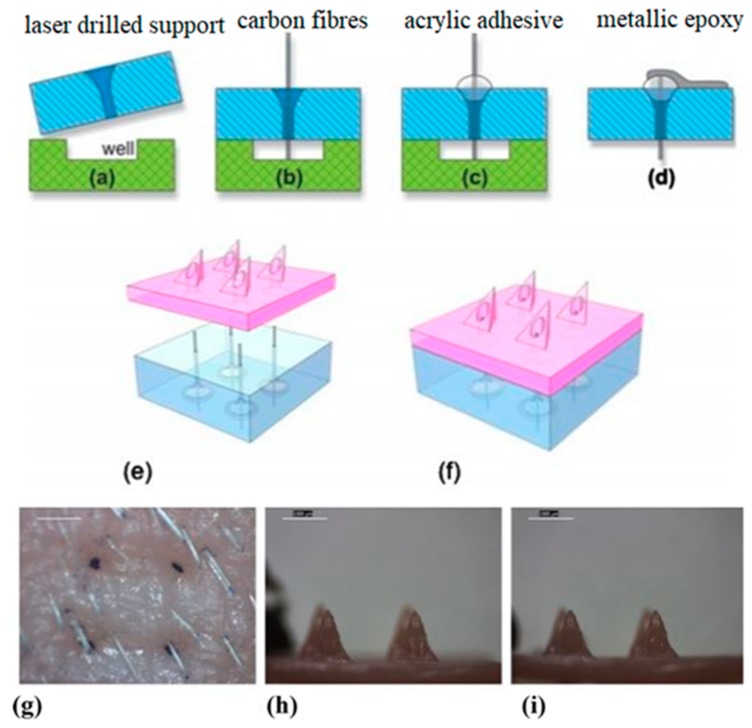
Schematic showing steps (**a**–**f**) used for assembly of the microneedle device. Images of microneedle array and cadaveric porcine skin after microneedle insertion. (**g**) Optical micrograph showing delivery of trypan blue into microneedle-fabricated pores within cadaveric porcine skin scale bar 1 = mm. (**h**) Optical micrograph showing hollow microneedles before insertion into cadaveric porcine skin. (**i**) Optical micrograph showing hollow microneedles after insertion into cadaveric porcine skin [42].

Miller et al. created polymer microneedle arrays with integrated carbon fiber electrodes for electrochemical sensing of biological analytes (e.g., ascorbic acid and hydrogen peroxide) [42]. The microneedles were triangular pyramids in shape, with base lengths of ~1120 µm, heights of ~1030 µm, and circular bores of ~375 µm (Figure 14). Penetration by the microneedle arrays into the stratum corneum layer of excised porcine skin was confirmed by the delivery of trypan blue dye. Electrochemical experiments involving the carbon-fiber electrodes within the hollow microneedles confirmed that ascorbic acid and hydrogen peroxide values could be determined. The carbon fibers were functionalized to enable detection of the analytes. The fibers were activated in KOH at 1.3 V (vs. Ag/AgCl); they were subsequently exposed to 2-amino-4-nitrophenol and sodium nitrite to create diazonium salt in situ. They were then scanned with cyclic voltammetry (CV) (0.4 V to −0.8 V, vs. Ag/AgCl) to graft the 2-nitrophenol and reduce it to aminophenol; for hydrogen peroxide detection, the fibers were exposed to palladium (II) chloride at a potential of −0.8 V (vs. Ag/AgCl). Electrochemical detection indicated dose-response behavior of the sensor for hydrogen peroxide, with a linear range between 100–500 µM and a predicted detection limit of 15 µM; for the ascorbate analyte, detection of a 1 mM concentration was possible at 195 mV.

**Figure 15:**
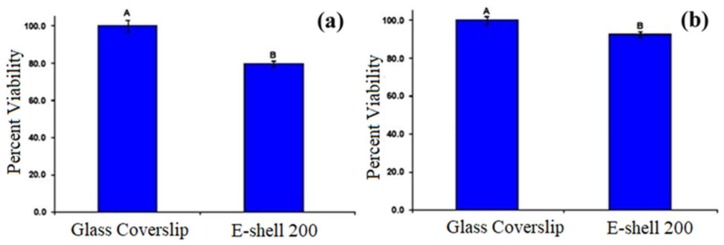
MTT viability data for cells grown on e-shell 200 acrylate-based polymer and glass cover slip. (**a**) MTT viability of human epidermal keratinocytes grown on e-Shell 200 acrylate-based polymer compared to glass cover slip. A and B denote statistical differences *p* < 0.05 between the polymer and the control. (**b**) MTT viability of human dermal fibroblasts grown on e-Shell 200 acrylate-based polymer compared to glass coverslip. A and B denote statistical differences *p* < 0.05 between the polymer and the control [42].

In addition, the e-shell 200 acrylate-based polymer underwent MTT assay testing with neonatal epidermal keratinocytes and human dermal fibroblasts (Figure 15). The results showed a significant decrease in the viability of the cells grown on the polymer compared to the control; however, the decrease in viability was not at levels that would be of concern for in vivo transdermal sensing applications.

Windmiller et al. fabricated triangle-based pyramidal microneedle arrays with hollow pores, and packed the hollow bores with metalized carbon paste (functionalized with rhodium and lactate oxidase); the ability of these electrochemical sensors to detect lactate was assessed. Three-by-three arrays of the pyramidal microneedles (1500 µm tall, 425 µm bore) were fabricated out of the e-shell 200 acrylate-based polymer using a dynamic light micro stereolithographic rapid prototyping system [43]. Electrochemical characterization of the electrodes in hydrogen peroxide solutions showed good linearity in the 0–500 µM range (r^2^ = 0.999) with a lower detection limit of ~20 µM; highly repeatable results were also observed with repacking of the carbon paste into bores of the microneedles following characterization.

Examination of lactate detection showed high linearity over (r^2^ = 0.990) a range of 0–8 mM lactate solutions, with an estimated detection limit of 0.42 mM lactate; this value is well below physiologic levels. In addition, lactate was able to be detected under challenge conditions with uric acid, citric acid, and acetaminophen as competing analytes with minimal deviation from expected values. The results of this study show progress toward an amperometric sensor for transdermal sensing of bioanalytes.

Electrochemical characterization of the electrodes showed linearity (r^2^ = 0.995) over a range of glutamate concentrations (0–140 µM) in buffer, with a sensitivity of 7.129 nA/µM and a predicted limit-of-detection of 3 µM; this limit is well-below the value observed physiologically. Additionally, linearity (r^2^ = 0.992) was observed over the same concentration range (0–140 µM) when the electrodes were tested in human serum; the limit-of-detection under these conditions was noted to be 21 µM with a sensitivity of 8.077 nA/µM. Glucose testing in buffer was also successful with linearity (r^2^ = 0.996) over the range of 0–14 mM; a sensitivity of 0.353 nA/µM and a limit-of-detection of 0.1 mM were noted. In addition, interference studies involving uric acid, ascorbic acid, cysteine, and acetaminophen during glutamate and glucose testing showed minimal interference by these competing analytes.

## 7. Transdermal Alcohol Sensors

**Figure 16:**
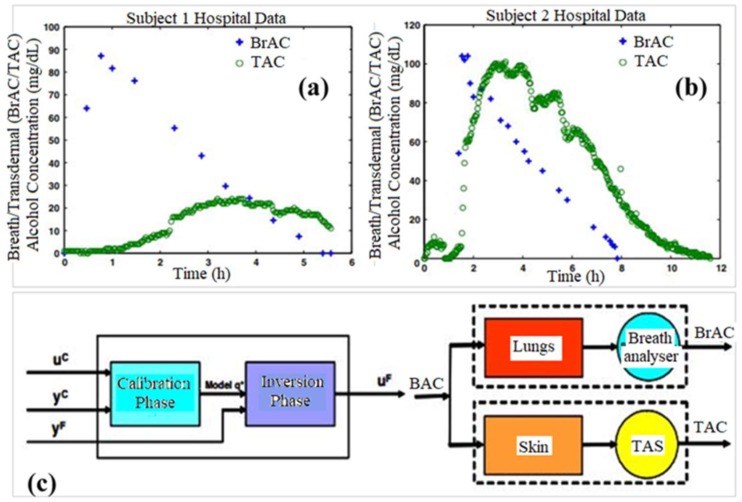
Breath and transdermal data for (**a**) Subject 1 and (**b**) Subject 2. (**c**) Two phase design of the data analysis system (left) and the blood/lung/breath analyzer and blood/skin/TAS systems (right) [44].

Dumett et al. described a mathematical modeling approach for determining blood alcohol concentrations (BAC) from transdermal alcohol concentrations (TAC); they compared this information to breathe alcohol concentration (BrAC) data [44]. Since ethanol can be detected in perspiration, a noninvasive transdermal sensor [45,46,47,48,49] for ethanol known as the WrisTAS^TM^ (Giner, Inc., Newton, MA, USA) has been developed. This device serves essentially as a sobriety check and does not provide quantitative data on the blood alcohol concentration of the individual wearing the device. The authors described a mechanism for comparing TAC measures obtained clinically from this type of device to BrAC values in order to determine quantitative BAC levels from TAC measurements (Figure 16). This process took into account (a) movement of alcohol through the skin and to the sensor, (b) calibration procedures for both the individual subject and the sensor being worn, and (c) deconvolution of the BAC value from the TAC measurement. An inversion scheme was used to test the ability of the model to accurately predict BrAC values based on TAC values. The approach was a good predictor of BrAC values from TAC measurements. Advances in this approach may allow BAC values to be obtained from TAC measurements. 

## 8. Ultrasound Technique for Transdermal Biosensing

Lee et al. examined transdermal sensing of glucose on the abdominal skin of rats, following permeablization of the skin with ultrasound [50]. A cymbal ultrasound array was applied to the abdominal area of the rats, delivery a low frequency (20 kHz) signal; this approach aided in the permeablization of the skin for sampling of interstitial fluid. 

**Figure 17:**
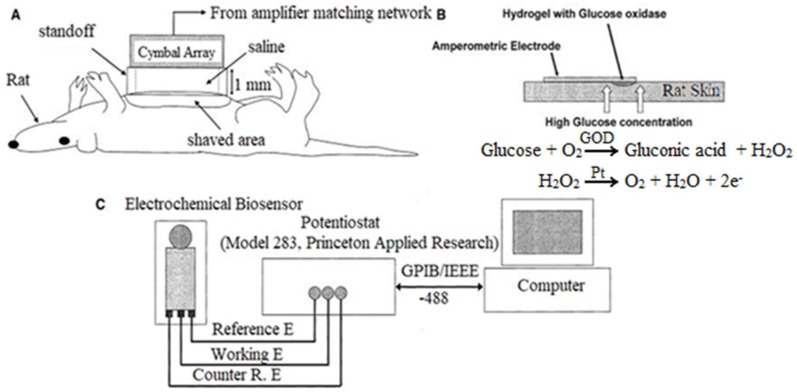
(**a**) Rat on its back; 1-mm thick water-tight stand-off between the shaved abdomen and array. Reservoir inside the stand-off filled with saline. US (ISPTP = 100 mW/cm^2^) applied to exposed group for 20 min; US not applied to the control. (**b**) In presence of glucose oxidase, glucose becomes gluconic acid via glucono-δ-lactone: hydrogen peroxide is generated as byproduct. Electrochemical biosensor uses the signal caused by oxidation of hydrogen peroxide. One glucose molecule generates an oxygen molecule, a water molecule, and two electrons in the reaction. (**c**) Voltage between working electrode and reference electrode, 0.7 V, applied from potentiostat controlled by computer. At the same time, current between two electrodes is determined by potentiostat and recorded in the computer [50].

Following ultrasonication, a three-electrode (platinum as working electrode, carbon as counter electrode, silver/chloride as reference electrode) cell with a glucose oxidase functionalized working electrode and a poly(ethylene glycol) hydrogel application pad was applied to the skin to detect glucose levels in the interstitial fluid of the permeablized area. Comparison of measurements obtained using the ultrasound/biosensor and a commercial glucose meter in hyperglycemic rats (Figure 17) showed no statistically significant differences between the measurements (356.0 ± 116.6 mg/dL and 424.8 ± 59.1 mg/dL, respectively); glucose could not be accurately detected by the biosensor without ultrasonication. Histology indicated tissue damage at high intensities (200 and 300 mW/cm^2^, 20% duty cycle) and no tissue damage at low duty cycles (100 mW/cm^2^, 20% duty cycle); this problem can be addressed by reducing the duty cycle to 15% or lower for higher intensities.

Smith et al. investigated ultrasound permeabilization of rabbit skin for transdermal delivery of insulin and transdermal biosensing of glucose levels in the interstitial fluid [51]. 2 × 2 and 3 × 3 cymbal ultrasound arrays were used to ultrasonicate (I = 100 mW/cm^2^, 20% duty cycle, 20 kHz) the abdominal skin of hyperglycemic New Zealand White rabbits. 

**Figure 18:**
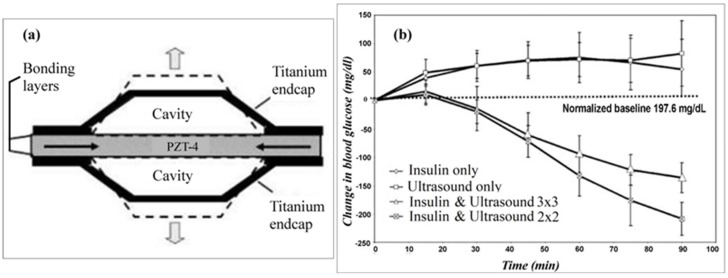
The cymbal transducer made of piezoelectric material lead zirconate-titanate (PZT)-4 operated at a frequency of 20 kHz. The cymbal disk was placed between two titanium caps with air cavities beneath the caps, which convert the radial oscillations of the disk into flexure motions of the caps. Graph of the change in blood glucose over the 90-min transdermal insulin delivery experiment duration, comparing both controls (insulin only and ultrasound only) to the insulin and ultrasound results generated by the 2 × 2 array (insulin and ultrasound 2 × 2) and 3 × 3 array (insulin and ultrasound 3 × 3) [51].

The insulin delivery process involved ultrasonicating a reservoir of 50 U/mL insulin in saline into the skin of the animal. Blood glucose measurements obtained using a standard glucose meter from the 2 × 2 cymbal array groups showed that after 60 min the blood glucose levels decreased at 30, 60, and 90 min time points (-20.0 ± 20.8 mg/dL, −132.6 ± 35.7 mg/dL, −208.1 ± 29 mg/dL, respectively) from the initial measured value of 245.4 ± 45.5 mg/dL; similar results were observed in the groups treated with the 3 × 3 cymbal array (Figure 18). However, the saline-only sonication group and the insulin without sonication group saw increase in blood glucose levels over the same time period. Noninvasive glucose sensing of the hyperglycemic rabbits was also conducted following ultrasonication; measurements obtained using this method were compared to those obtained using a standard glucose meter (Figure 17). 

These experiments were conducted with a three-electrode (platinum as working electrode, carbon as counter electrode, silver/chloride as reference electrode) cell using a glucose oxidase-functionalized working electrode and a poly(ethylene glycol) hydrogel application pad. The results indicated that the measured values of blood glucose from the noninvasive ultrasound method were comparable to the conventional glucose meter (395 ± 42 mg/dL and 425 ± 35 mg/dL, respectively); the results also indicated that the noninvasive glucose sensor was unable to accurately detect glucose levels without ultrasonication. 

Li et al. proposed a minimally invasive technique to determine glucose levels that coupled ultrasound application to permeabilize the skin, vacuum extraction of interstitial fluid, and determination of the glucose concentration in the interstitial fluid by surface plasmon resonance [52]. The authors proposed permeabilizing the skin by breaking down the stratum corneum with low-frequency ultrasound (55 kHz), enabling transport of interstitial fluid.

The interstitial fluid could then be extracted by vacuum at <10 in. Hg in 20-min intervals (15 min needed per extraction) for up to 15 h (the projected length of time over which the skin maintains enhanced permeability). They developed a method to predict blood glucose levels based on glucose levels in the interstitial fluid with a prediction error of 16%. Following collection of the interstitial glucose, the samples could be analyzed with surface plasmon resonance, calibrated to detect the changes in the reflective index of the solution as due to glucose. Using this technique, the authors established the ability to detect glucose at 10 mg/dL. They are also investigating the coupling of glucose to proteins in a protein-glucose binding method (e.g., d-glucose/d-galactose-binding protein) for enhancing the sensitivity of the technique; this approach involves binding glucose onto the sensor of the surface plasmon resonance detector.

## 9. Microfluidics Approach in Transdermal Sensing

**Figure 19:**
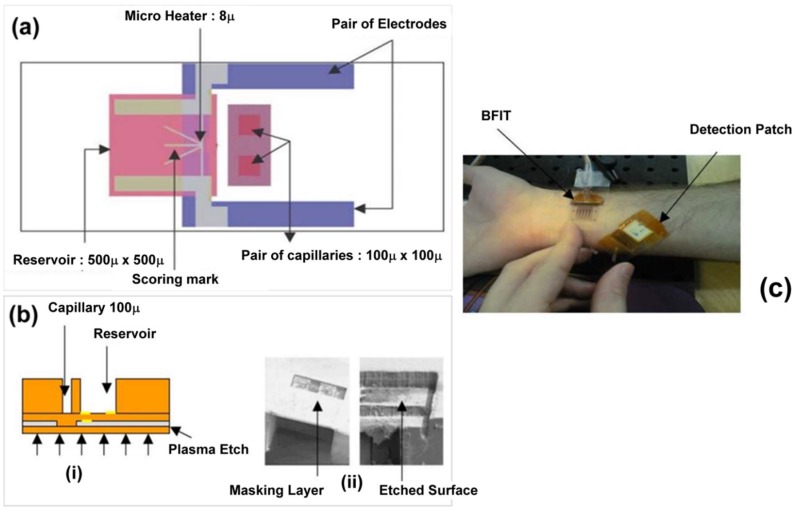
(**a**) A single B-FIT device cell with all masking layers. (**b**-(**i**)) Cross-section of the released device, (**b**-(**ii**)) SEM micrographs of SU-8 dry etching using masking layer. (**c**) Fully functional B-FIT pictured on human arm [53].

Gadre et al. demonstrated a novel fabrication strategy to create an integrable transdermal (B-FIT) microsystem for detection of biomolecules in interstitial fluid [53]. The B-FIT system was incorporated into a robust dermal patch with complex microfluidics, a heater element, and a colorimetric detection membrane. The fabrication process involved deposition of multilayers of SU-8 epoxy-based photoresist onto a glass slide with a Teflon release layer and a sacrificial aluminum layer for preparing microfluidic capillaries and the fluid reservoir (Figure 19). Sputtering, lithographic patterning, and chemical wet etching were utilized to produce the chromium/gold electrodes of the micro-heater. The B-FIT heater elements enable thermal ablation of micropores in the upper layers of the skin for diffusion of biomolecules to the transdermal sensor. Nisar et al. applied a three-dimensional model with coupled field effects for finite element analysis of the piezoelectric and fluidic performance of a proposed micropump for transdermal drug delivery-based treatment of cardiovascular disorders [54]. The valveless, PDMS pump design contains diffuser and nozzle elements and is driven by a piezoelectric actuator. Piezoelectric actuator deflection and micropump flow rate were evaluated at various operational parameters with varying voltage and excitation frequency both experimentally and theoretically.

The highest flow rate of the micropump was measured experimentally using deionized water; it was approximately 8.9 μL/min at 160 Vp-p with an excitation frequency of 250 Hz. The experimental and numerically predictive values of micropump flow rates were similar, validating the transient multifield analysis model. The results demonstrated that the flow rate is more dependent on the excitation voltage than the excitation frequency; furthermore, a high excitation voltage at constant frequency was shown to be important for an increased flow rate. Other microfluidic systems have also been designed for efficient and targeted drug delivery [55,56]

## 10. Optical Transdermal Sensors: A Condensed Overview

### 10.1. Fluorophore Based Glucose Sensor

Lakowicz et al. adopted the polarization approach for glucose sensing using a stretch-oriented reference film of Hoechst dye in polyvinyl alcohol and a fluorophore-based sensor containing a polarizer [57]. The polarizer located in the sensor was oriented perpendicularly to the sample and the excitation source; this approach ensured sample emission added only to the perpendicular total intensity. The glucose-sensitive fluorescence signal was emitted by mutant glucose/galactose binding protein (GGBP) from E. coli with a single cysteine residue inserted at position 26 and labeled with 2-(4′-iodoacetamidoanilino)naphthalate-6-sulfonic acid (I-ANS). Two excitation sources were utilized, including a UV hand lamp for excitation of the sample and a spectrofluorometer for excitation of the reference film. Polarization sensing of glucose was demonstrated with an accuracy of 0.2 μM glucose concentration; polarization increased with increasing glucose levels.

**Figure 20:**
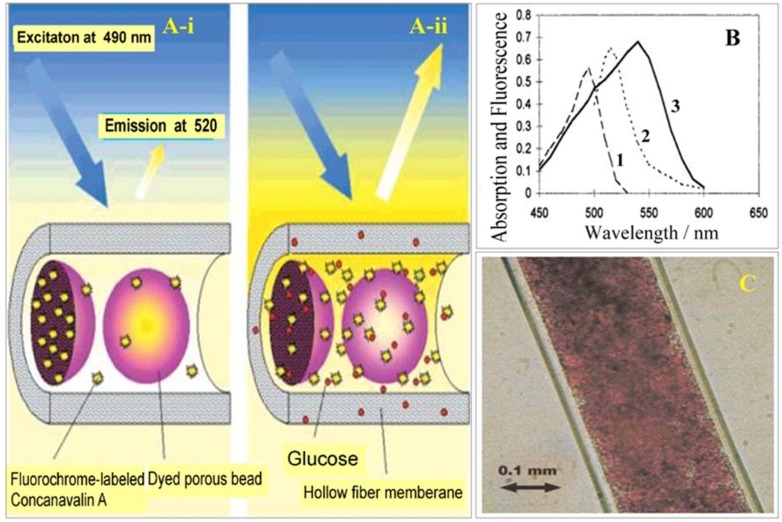
(**a**) Schematics illustrating the principles of the fluorescence affinity hollow fiber sensor. In the absence of glucose, fluorochrome-labeled Concanavalin A is bound to the fixed glucose residues inside the porous beads (**A-i**). The beads are colored with dyes that prevent the excitation light from inducing Con A to fluoresce, keeping the fluorescence emission at 520 nm. After diffusion of glucose through the hollow fiber membrane (molecular weight cutoff, 10 kDa), Con A is displaced from the beads and diffuses out of them. Fluorochrome-labeled Con A becomes exposed to excitation light, resulting in a strong increase in fluorescence (**A-ii**). (**b**) Spectra of fluorescence and absorption of the various chromophoric components of the bead-based affinity sensor. (1) Excitation spectrum of fluorescein, (2) emission spectrum of fluorescein, (3) absorption spectrum of dye-labeled Sephadex beads. (**c**) Light microscopy picture of a small section of the hollow fiber that was filled with dye-colored Sephadex G150 beads. A bead fraction having a bead diameter of less than 25 µm was used in this study; the beads were obtained by sieving the original material (mesh size of 25 µm) [58].

Ballerstadt et al. described a fluorescence hollow fiber glucose sensor with porous dyed Sephadex beads containing immobilized glucose residues and Alexa488-labeled concanavalin A (Figure 20) [58].

The hollow fiber dialysis membrane contains 20–50 um Sephadex beads dyed with safranin O and pararosanilin. In the presence of glucose, Alexa-488-ConA molecules are displaced from the beads and exposed to excitation light, resulting in an increase in fluorescence emission at 520 nm. The sensor demonstrated a rapid response time, a glucose detection range from 0.15 to 100 mM, and a dynamic signal change from 0.2 to 30 mM; however, stability studies indicated a 40–50% reduction in sensor intensity over three months [58]. Ballerstadt et al. incorporated a near-infrared chromophore linked to ConA within an immobilized bead matrix [59]. The fluorophore Alexa 647 was utilized to extend the fluorescence emission of the glucose sensor to the near-infrared region (670 nm), enabling transmission through the skin for transdermal glucose monitoring. A six-month study showed that the glucose sensitivity of the sensor gradually decreased over time; it should be noted that the sensor response amplitude for a glucose concentration of 2.5–10 mM remained stable. Ballerstadt et al. further evaluated the long-term stability of the near-infrared affinity sensor under physiological conditions [60]. The sensor was subjected to in vitro testing using continuous glucose cycling with alternating exposures to glucose concentrations of 2.5 and 20 mM for three-hour segments at 37 °C. The long-term performance of the sensors showed an initial increase in fluorescence during the first 3–4 weeks of the study, followed by a gradual and linear decrease. The authors suggest that leakage of Alexa6470-ConA is the underlying cause for the 60% loss in the fluorescence signal over the course of the long-term study. They also performed acute and chronic in vivo tests in hairless rats using a cellulose hollow fiber containing a suspension of Cy7-succinimidyl ester-ConA-Sepharose, Alexa647 dextran, and TransFluoSpheres with 2 µm diameter [61]. The sensor demonstrated extremely high sensitivity for a glucose concentration around 5 mM and between 20–25 mM. At two weeks post-surgery, the implanted sensors exhibited 10–15 min delays but were able to detect variations in blood glucose levels. Minimal host response was observed for the implanted sensors. In addition, intradermal injections of the sensor components in rats showed no toxicity compared to the control group. 

McShane et al. developed a custom fiber optic probe and optical system for delivery and collection of light to an implanted fluorescence-based sensor for glucose monitoring in interstitial fluid [62]. The system was compared with two commercially-available fluorometers and demonstrated a higher peak response and a faster time per scan than both commercial instruments. Solutions of FITC-dextran and TRITC-Con A at different ratios were measured under similar conditions upon incremental addition of glucose. The system observed high fluorescence measurements from implanted fluorescence particles in both excised porcine skin and live animals; however, quantitative measurements were not possible due to fluctuations in the implantation depth. 

Long et al. fabricated microsphere glucose sensors that contained encapsulated glucose oxidase, catalase, and platinum octaethylprophine (PtOEP) in silica-alginate microspheres, which were coated with an ultrathin polyelectrolyte film [63]. These luminescence-based microparticle sensors were produced with various concentrations of enzymes and evaluated using a flow through system with three different glucose concentrations (0, 25, and 75 mg/mL). 

The particle sensors were embedded in a PEG hydrogel. The glucose levels were maintained for a duration of 8 h and repeated in triplicate. Theoretical optical modeling of the sensors suggested the emission signals from the particles are adequate for high signal-to-noise ratio measurements. Experimental evaluation of the microparticle sensors revealed no significant difference in sensor stability with increasing catalase concentration; in addition, trace amounts (≤100 µM) of catalase in commercial glucose oxidase were sufficient for stabilization of the enzyme.

**Figure 21:**
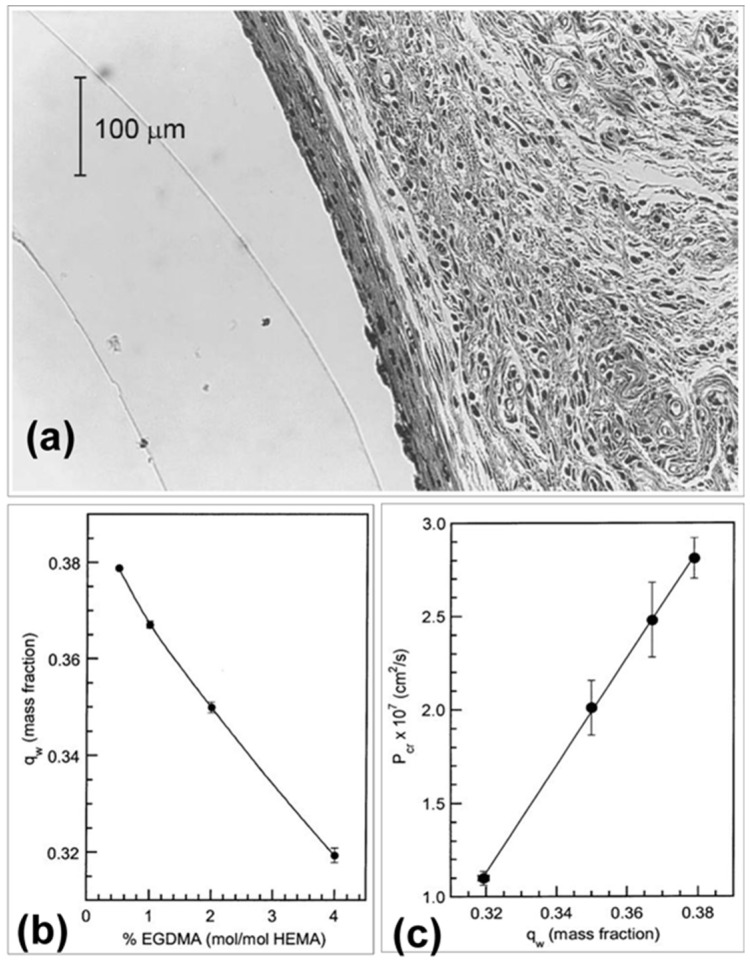
(**a**) Photomicrograph of the host response to a pHEMA membrane implanted for 3 weeks. (**b**) Dependence of equilibrium water content, q_w_, for flat sheet pHEMA membranes at 37 °C on crosslinking ratio. Experiments were performed in triplicate at each crosslinking ratio. (**c**) Dependence of creatinine permeability at 37 °C in flat sheet pHEMA membranes on equilibrium water content. For reference, the permeability of creatinine in a 1000 MWCO cellulosic dialysis membrane is 9.0 × 10^−7^ cm^2^/s [64].

Kermis et al. evaluated the permeability properties of optically transparent ethylene glycol dimethacrylate (EGDMA) crosslinked poly(2-hydroxyethyl methacrylate) (pHEMA) membranes for transdermal glucose detection (Figure 21) [64]. The transport properties of the flat sheet and cylindrical membranes were determined using a diffusion cell at 37 °C. The creatinine and IgG-Fab fragment concentrations were determined using a UV-Vis diode array spectrophotometer and a digital fluorometer. The permeability experiments indicated that an increase in crosslinker concentration decreased the degree of membrane swelling, which resulted in lower creatinine permeability and increased selectivity for the protein. In vivo studies in rats showed that subcutaneous implantation of the 4% EGDMA cylindrical membranes up to six weeks resulted in minimal host response with a thin fibrotic capsule and negligible loss of membrane integrity. 

Kholodnykh et al. demonstrated glucose monitoring using optical coherence tomography (OCT); glucose-induced changes in the optical signal attenuation could be detected with an implantable turbidity sensor [65]. The sensor contained cellulose membranes encapsulating a suspension of ConA and hydrogel particles ranging from 1–4 µm in diameter; the sensor also included a highly specularly reflective Mylar film. They measured changes in light attenuation as a function of glucose concentration; they determined that an increase in glucose level was associated with a decrease in the optical turbidity of the sensor. In vitro testing using excised skin tissue demonstrated the advantages of measuring specular reflection over backscattering light attenuation. When testing the sensor covered by tissue phantoms, the specular reflection decreased almost linearly with increasing implantation depth; the lowest acceptable sensitivity of 1 bD per mM glucose was estimated at an optical depth of 12 mean free paths (mfp) which translates to approximately 1.2 mm of skin tissue and a scattering coefficient of 10 mm^−1^. 

Khan et al. developed five different mutants of glucose/galactose-binding protein (GBP), fluorescently labeled the mutants with the fluorophore 6-bromoacetyl-2-dimethyl aminonaphthalene (Badan), and evaluated the GBP mutant response to glucose [66]. An emission wavelength of 550 nanometers and an excitation wavelength of 400 nanometers were used for steady-state fluorescence measurements of the labeled proteins. The triple mutant H152C/A213R/L238S-Badan demonstrated the highest binding constant of 11 mM in PBS and 14 mM in serum; the change in fluorescence intensity upon the addition of glucose was 180% in PBS and a 200% in serum. This mutant was capable of glucose sensing within the physiologically relevant range of 1–100 mM. Potential applications of the GBP mutant-based fluorescence sensor including use for continuous in vivo monitoring of glucose concentrations in diabetic patients. 

### 10.2. Hydrogel-Based Fluorescent Glucose Sensors

**Figure 22:**
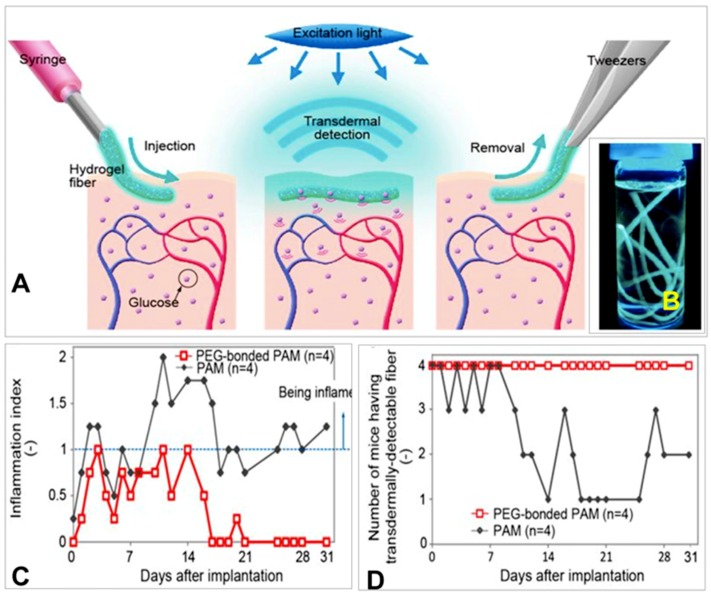
(**A**) Schematic illustration of the fluorescent hydrogel fiber designed for long-term in vivo glucose monitoring. The fiber can be injected into subcutaneous tissues. The implanted fiber remains at the implantation site for a long period and transmits fluorescent signals transdermally depending on blood glucose concentration. The implanted fiber can be easily removed from the implantation site after use. (**B**) Fluorescent hydrogel fibers in a glass vial with a 50% glucose solution. The fibers are excited by ultraviolet light. The fluorescent image indicates that the glucose-responsive monomer is immobilized within the hydrogel fibers. (**C**) Inflammation indices of the mouse ears with the fibers one month after implantation. Inflammation was evaluated based on reddening, swelling and scab formation for a month. The inflammation index was obtained by summing the reddening, swelling, and scab formation scores. If the ear skin showed any reddening, reddening scores 1 point; similarly, swelling and scab formation were each also scored 1 point. PEG-bonded PAM induced less inflammation than PAM only. (**D**) Numbers of mice showing transdermal transmission of fiber fluorescence [67].

Heo et al. applied a fluorescent polyacrylamide (PAM) hydrogel and polyethylene-glycol (PEG)-bonded PAM hydrogel fibers for long-term in vivo glucose sensing [67]. The fibrous structure of the fibers permitted good adhesion to the tissue with minimal migration, enabling the sensor to remain in the implant site. The high surface area to volume ratio of the fibers with approximately 1 mm diameter enabled high diffusion for rapid response times. For in vitro studies, the fibers were exposed to various glucose concentrations covering the physiological normal, hypoglycemic, and hyperglycemic ranges (0–500 mg/dL). Fluorescent images were obtained at a wavelength of 488 nm; both fiber types demonstrated increasing fluorescence intensity with increasing glucose concentration (Figure 22). In vivo evaluation in the transparent ear skin of mice indicated 75% of the PEG-bonded PAM hydrogel fibers responded to blood glucose concentration fluctuations. Only 25% of the PAM hydrogel fibers responded to the glucose changes. A ten-minute delay was observed between blood glucose concentration change and fiber response. 

**Figure 23:**
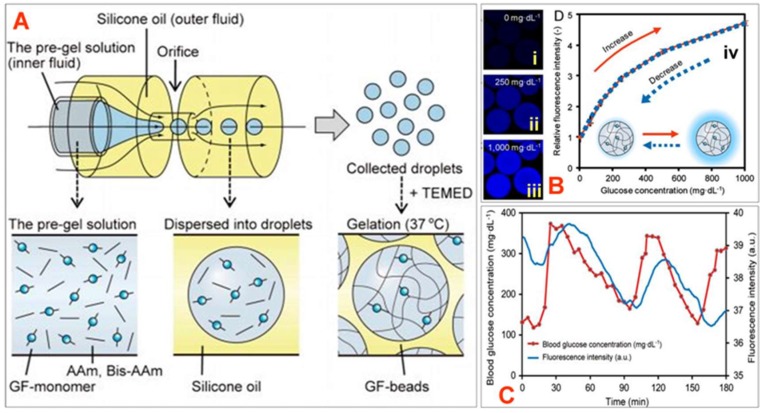
(**A**) Fluorescent microbeads fabricated by a microfluidic system. (**A**) Schematic diagram of the AFFD. The pregel solution flows into the inner channel of the AFFD, and the silicone oil flows into the outer channel. The droplets in silicone oil are collected from the solution with TEMED at 37 °C; they turn into microbeads after gelation. (**B**) Glucose responsiveness of GF-beads. (i–iii) Fluorescent images of GF-beads at a glucose concentration of 0 mg·dL^−1^, 250 mg·dL^−1^, and 1000 mg·dL^−1^, respectively, (iv) The fluorescence intensity of GF-beads (n = 19) changes according to the increase and decrease in glucose concentration. The overlapping curves are evidence of the reversible reaction between GF-beads and glucose. (**C**) The fluorescence intensity traces blood glucose concentration. The time lag mainly comes from the time response between the interstitial fluid glucose concentration and the blood glucose concentration [68].

Shibata et al. developed highly sensitive and injectable microbeads containing a glucose-responsive fluorescent monomer for in vivo continuous glucose monitoring (Figure 23) [68]. The fluorescent monomer was derived from a glucose-responsive dye, which was comprised of a diboronic acid moiety and an anthracene moiety; polyethylene glycol and polyacrylamide groups served as spacers.

Glucose responsiveness testing of the microbeads indicated that the fluorescence intensity increased with increasing glucose concentration (0–1000 mg/dL); the association was found to be reversible since the intensity curves matched with an increase and decrease in glucose concentration. In vivo implantation of the microbead sensors in the ear skin of mice showed that the microbeads were able to monitor fluctuations in blood glucose concentration with a lag time of 11 min.

### 10.3. Fluorescence-Based Ion Sensors

Geddes reviewed the medical relevance of halide ion sensing in a variety of diagnostic processes [69]. Monitoring of fluoride levels can diagnosis high fluoride levels in the blood; while fluoride is particularly present in the teeth and bone, its slow clearance from the body following excessive intake can result in poisoning. Additionally, determining toxic levels of bromide (an unnecessary or misunderstood ion of the trace element in the body) and biological levels of iodide (which is important in production of certain hormones) have medical significance.

Levels of fluoride, chloride, bromide, and iodide ions in blood, urine, sweat, and serum have been tested for diagnostic purposes using a variety of testing methods; these methods include use of ion selective electrodes, ion chromatography, clinical analyzers, microvolume methods, titrator strips, gas chromatography, anion-exchange chromatography, high-purity liquid chromatography (HPLC), head-space flow injection, x-ray fluorescence, and HPLC with Ag electrode. However, the fluorescence quenching behavior of fluorophores by halides has been investigated as an alternative sensing method to those previously listed; this property may be incorporated into transdermal sensing techniques. Fluorescence occurs due to the rapid emission of a photon when an electron in an excited orbital is allowed to return to the ground state quickly (the electron is “spin allowed,” by having the opposite spin to the ground state electron with which it is paired). Quenching of this fluorescence signal is the basis for optical halide sensing. 

Fluorescence from different marker agents (e.g., quinine, methoxyquinoline, rhodamines) may be quenched in the presence of halides, with the decrease in intensity or length of the fluorescence signal being correlated to halide concentration. The process of dynamic fluorophore quenching by halides was first described by the Stern-Vollmer kinetics; more recently, the kinetics have been extended to determine the effects of additional quenching halides (e.g., a solution containing I^−^, Cl^−^, Br^−^) by adding terms to the equations. Similarly, the Stern-Volmer calculations can be slightly modified to account for scenarios where there is static quenching of the fluorophore or limited accessibility to the fluorophores due to multiple fluorophore populations (e.g., the halide can quench one fluorophore on a molecule, but cannot reach the others). Another aspect of fluorophore quenching that can be harnessed for detection is fluorescence resonance energy transfer (FRET); in this detection technique, the energy of an electron in an excited state is donated to an acceptor molecule; this molecule has an excitation spectrum that overlaps with the emission spectrum of the donor. By monitoring this dipole-dipole interaction fluorescence in the presence and absence of the solvated halide-ion of interest, detection of the halide ion can occur. Other factors to consider with the halide ion detection via fluorescence quenching are dissolved oxygen concentration (which in itself can act as a quenching agent) and the electrostatic charge of the environment surrounding the fluorophore (which can affect charged quenching agents). By utilizing these properties of fluorescence quenching by halide ions, optical sensors can be created to gauge the concentrations of halide ions of interest by immobilizing fluorophore molecules onto or within the sensor. The quenching of the fluorophore molecules when interacting with the halide ions allows for the detection of the halide ions. Fluorophores such as acridinium, quinolinium, and rhodamines have been utilized in this manner as sensor detection molecules; similar sensors can be created by immobilization of FRET donor and acceptor molecules, allowing for the changes in fluorescence quenching in the presence of the halide ions. The potential for multiphoton detection of halide ion concentrations may also be possible for transdermal sensors. By utilizing an implanted halide ion sensor under the skin, excitation by multiphoton laser light (e.g., femtosecond pulses at λ = 800 nm), fluorescence detection may be accomplished within the “therapeutic range” of 600–1000 nm; the auto-fluorescence interference associated with tissues at physiologically relevant concentrations of the halide ions of interest can be avoided. This technique harnesses the energy of multiple photons of light at a focal point during a short time period (10^−15^–10^−16^ s) to excite the fluorophore that would otherwise need the energy of a shorter wavelength of light to emit a photon during relaxation of its excited electron. By utilizing the combined energy of the excitation photons at the focal point, a longer wavelength of light can be used that is less damaging to, and avoids interference from, biological fluids and tissues.

**Figure 24:**
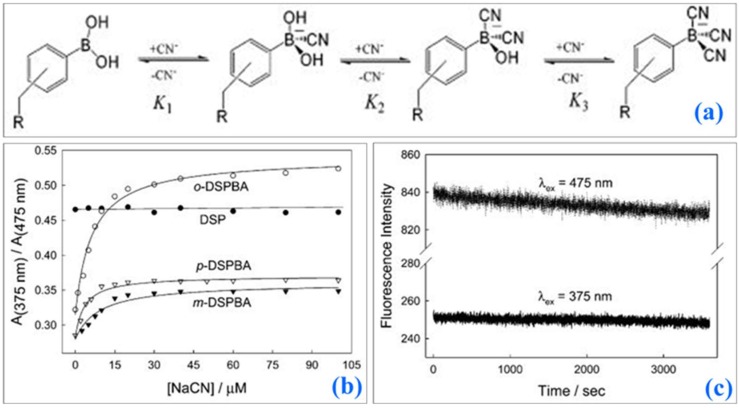
(**a**) Complexation of DSPBA probes with aqueous free cyanide. (**b**) Ratiometric response of the probes in water with increasing cyanide concentrations. (**c**) Fluorescence emission of o-DSPBA in water with time when excited at 475 and 375 nm [70].

Badugu et al. demonstrated the cyanide detection up to physiologically relevant levels using water-soluble fluorescent probes (Figure 24). Three isomeric boronic acid derivative probes, *ortho*-, *meta*-, and *para*-(4-[4-(*N*,*N*-dimethylamino)styryl]-1-(*x*-boronobenzyl)pyridinium bromide (DSPBA), were bound to cyanide resulting in a reduced intramolecular charge transfer for enhanced fluorescence sensing. The *ortho*-DSPBA isomer probe demonstrated the best response to cyanide compared to the other isomeric probes due to improved electrostatic interactions.

Cyanide concentrations of 1–30 µM were detectable in the presence of physiologically relevant interferants, such as aqueous chloride up to 100 nM. The emission of the probes fell within the optical window of tissue and blood thus demonstrating the sensor applicability in transdermal cyanide monitoring. 

**Figure 25:**
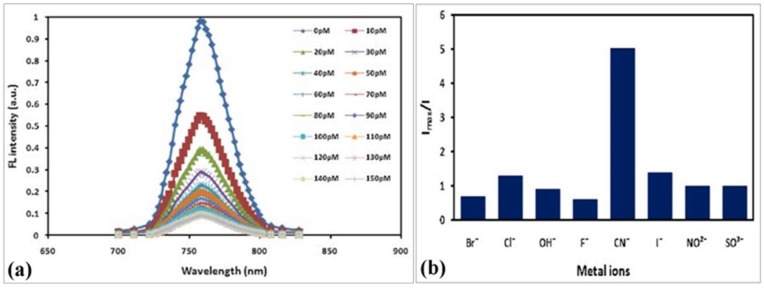
(**a**) FL intensity for functionalized InP QWs at different cyanide concentration. (**b**) Relative intensity for functionalized InP QWs for various metal anions at 50 pM concentration [71].

Another report demonstrates the detection of cyanide anions in solution has been accomplished by monitoring changes in fluorescence of indium phosphide quantum wires (InP QWs) that have been coated with mercaptohexanoic acid (Figure 25) [71]. 

These quantum wires were modified to have a peak emission wavelength of λ = 750 (±10) nm (excitation λ = 375 nm), which is optically compatible with blood and tissue; these devices have potential use for transdermal detection of cyanide ions. The InP QWs exhibited dose-dependent linear decreases in fluorescence, potentially due to Förster resonance energy transfer between the InP QWs and the cyanide ions, upon exposure of the QWs to cyanide ions down to picomolar concentration levels (range of 0–110 × 10^−12^ mol L^−1^). The InP QWs also showed a limited response to potentially interfering ions, indicating specificity to CN^−^.

### 10.4. Luminescent Sensors

Long et al. further predicted the performance of these implantable luminescent sensors using three dimensional, multiwavelength Monte Carlo modeling to determine the distribution of the emitted luminescence at different implantation depths, excitation light source characteristics, particle properties, and particle packing efficiency [72]. The modeling system enabled realistic simulation of light propagation in a four-layer skin tissue model with embedded microparticle sensors hexagonally packed at different densities. The spatial distribution of the emitted luminescence induced by an excitation beam with a diameter of 10 mm can be contained within an 18 mm circle, allowing a sensor design utilizing a matched optoelectronic system to effectively deliver the excitation source and subsequently collect and analyze the luminescence. The simulation study indicates a strong dependency of signal attenuation on the depth of implantation, with the predicted ratio of output to input power ranging from 10^−3^ to 10^−6^ for sensors at the proximal and distal regions of the dermis. Analysis of the effect of sensor packing on absolute output showed a higher luminescence intensity with tightly packed microspheres, requiring fewer particles, suggesting a single site implantation with a high concentration of sensors.

### 10.5. Nanomaterial-Based Optical Sensors

Aslan et al. utilized dextran-coated gold nanoparticle colloids to measure wavelength-ratiometric resonance light scattering for transdermal dynamic glucose sensing [73]. Addition of glucose to the complex caused disassociation of the aggregates, resulting in an intensity decrease and a blue shift in the scattering spectrum. The ratio of scattered light intensities at different wavelengths (560 and 680 nm) by the nanogold aggregates was independent of aggregate concentration and light excitation. Absorption measurements were performed using a Varian UV/VIS 50 spectrophotometer; scattering measurements were conducted using a white LED light and a simple scanning monochromator. Based on measurements obtained by dextran-nanogold plasmon resonance, glucose concentrations were able to be determined in a sensing range of 1–60 mM. 

### 10.6. Raman Spectroscopy Based Transdermal Biosensors

The use of Raman spectroscopy for detection of biochemicals via transdermal biosensing has been explored [74]. The monitoring of blood glucose and interstitial glucose levels in diabetic patients is one example of this type of noninvasive monitoring. A glucose-specific Raman signature may be obtained from irradiance of the skin by a laser. Skin irradiance with an 830 nm wavelength laser light for 10 s at 0.36 W/cm^2^ is sufficient to obtain a Raman signal while remaining within safe operating parameters (ANSI 2000). Analysis of the vibration stretching band of the COH group at 2900 cm^−1^ or the stretch bands of COO and COC in the range of 900–1200 cm^−1^ can allow for detection of glucose [75]. Furthermore, the use of 830 nm near-infrared light can minimize biological auto-fluorescence that interferes with specific molecular signal recognition [76]. Subtraction of the interfering background fluorescence caused by biological fluids and skin can improve the detection of the target molecule (e.g., glucose) [76]. 

Avoidance of the interfering signal was attempted by Shao et al.; they focused the laser on a blood vessel for Raman detection of glucose and hemoglobin [77]. The hemoglobin served as an internal standard, and background contributions were determined from the tissue surrounding the in-focus blood vessel. The Raman signal peak at 1549 cm^−1^ for hemoglobin was compared to the most intense peak observed for glucose (1125 cm^−1^); the ratio of these intensities was used to determine the glucose concentration. While the authors were able to observe a concentration-dependent linear relationship for the intensity changes in the mouse model, the limit of detection for the system was 50 mmol/dl for testing the glucose solutions. This value is well above physiologically relevant blood-glucose levels (3–10 mmol/dL and <30 mmol/mL for health individuals and diabetic patients, respectively), indicating a need for an improvement in sensor detection capabilities. Incorporation of the surface-enhanced Raman scattering (SERS) effect, which is observed on nanostructured metallic materials, may also prove beneficial in transdermal Raman detection schemes. The SERS effect resulting from a target analyte adsorbed onto or in close proximity to a SERS substrate may increase the intensity of the signal by a factor of 10^3^–10^8^ [74,78]. 

The increased Raman signal is attributed to localized surface plasmon resonance effects that are caused by an electromagnetic enhancement of the Raman scattering [78,79,80]. Lyandres et al. demonstrated quantitative detection of glucose using the first in vivo surface-enhanced Raman spectroscopy (SERS) sensor [78]. A film over nanospheres (FON) structure was fabricated; an aqueous suspension of polystyrene nanospheres was deposited on a titanium substrate and coated with a 200 nm layer of silver. This structure was chemically functionalized with a self-assembled monolayer of 1-decanethiol and 6-mercapto-1-hexanol or benzenethiol (BZT). These SERS-active sensors demonstrated rapid and reversible interactions with glucose and high stability up to ten days with only a 2.08% decrease in intensity over time. In vitro studies using the Clarke error grid showed 85% accuracy for validation testing in the physiologically relevant range of 10–450 mg/dL glucose; results fell within the clinically acceptable range in the presence of interfering analytes. 

Additional studies showed that the sensors were able to track changes in glucose over time in rats; the trends measured with the SERS sensor were similar to those indicated by a commercial electrochemical sensor; the difference in measurements between the two approaches emphasizes the need for careful calibration and determination of accuracy. This design utilized subcutaneous implantation of the sensor, which was optically accessible through a glass window that was implanted above the sensor; this approach limited interference caused by skin tissue. This design was further improved by eliminating the glass window and capturing the signal from the SERS sensor transcutaneously with spatially offset Raman spectroscopy (SORS) [81]. SORS collects the scattered Raman signal from the concentric area surrounding the central laser excitation point rather than the signal at the excitation point [82], eliminating some of the interference caused by the skin surface; this approach also facilitates capture of the SERS signal from a deeper location. The device was able to track changes in interstitial glucose levels in a rat model as a proof-of-concept. A later iteration of the device showed improved accuracy and reliability to 17 days during in vivo testing in a rat model; only an initial calibration was required [83]. The measurements indicated that the root-mean-square error for calibration (RMSEC) and prediction (RMSEP) values for the device were below the ISO/DIS 15197 standard for the hypoglycemic range (<80 mg/dL); it is typically difficult to achieve accuracy over this range [84,85,86,87]. The accuracy and stability of the device indicate the potential use of this approach for continuous glucose monitoring.

Despite the challenges posed by various physiologic conditions that complicate in vivo Raman signal acquisition, there are a number of other target analytes for transdermal detection with Raman spectroscopy. Non-invasive detection of lactic acid levels in the blood or muscles of athletes is one possibility. Following calibration of a near-IR Raman setup for detection of lactic acid levels in human serum and Wistar rat blood, an optical fiber catheter was utilized to detect lactic acid levels transcutaneously in the ileac vein of Wistar rats after intraperitoneal injections of aqueous lactic acid solution (0.12 mol/L) [88]. An increase in Raman intensity was observed 30 s after injection, suggesting that lactic acid can be measured in this transdermal manner.

Skin cancer screening is another potential application of Raman spectroscopy. Optical identification of biomarkers in malignancies can (a) reduce the time needed for diagnosis and (b) minimize the pain and damage caused by unnecessary biopsies. Raman spectroscopy has been utilized in efforts to distinguish the differences between malignant and benign tissues. Basal cell carcinoma (BCC) lesions, squamous cell carcinoma (SCC) lesions, and inflamed scar tissues have been investigated in such a manner. Raman spectra obtained from scar tissue, BCC lesions, and SCC lesions in 19 patients (21 lesions) were compared to healthy sites on the patient skin [89]. A Raman probe was used to obtain 30 s signals from the lesions and the perilesional healthy tissue sites. Spectral analysis was able to correctly classify 100% (21/21 lesions) of the abnormal tissue sites as inflamed scar tissue, BCC, or SCC; it also identified 91% (19/21) of the healthy tissue sites. The changes in the spectral peaks associated with different lipid and protein structures caused by various disease states can be used for identification of the lesions and surrounding tissue. 

Another study, which spanned a little more than eight years, investigated in vivo Raman diagnosis of 518 skin lesions from 453 patients [90]. In addition to BCC and SCC lesions, the study investigated diagnosis of melanomas, actinic keratoses, atypical nevi, melanocytic nevi, blue nevi, and seborrheic keratoses. The Raman integration was completed within one second and utilized differences in the peak intensities in the spectral range of 500–1800 cm^−1^ in order to make a diagnosis; the 1055–1800 cm^−1^ wavelength range was noted as being useful for evaluation of melanoma. The study indicated that through use of principal component with generalized discriminant analysis (PC-GDA) and partial least-squares (PLS) analysis of the acquired Raman data, differences between malignancies and pre-malignancies from benign conditions, melanomas from nonmelanoma pigmented lesions, and melanomas from seborrheic keratoses can be identified with accuracies similar to those of clinical and other optical-based characterization methods. In a similar manner, the biochemical signatures of breast cancer lesions have been investigated via Raman spectroscopy. 

## 11. Miscellaneous Transdermal Biosensors

Lin et al. evaluated a continuous blood glucose monitoring system in female white rabbits [91]. The system contains three main components, including (a) a blood sampling and mixing mechanism with a peristaltic pump and an air stirring pump, (b) a glucose oxidase-based sensing probe (YSI Inc., Yellow springs, OH, USA.) for blood glucose measurements, and (c) a data acquisition network. 

An in vivo analysis methodically evaluated the effect of various parameters on the system such as (a) glucose concentration, (b) sampling pumping rate and duration, and (c) diameter of the withdrawing tube for blood and buffer samples. The glucose concentration measurement increased with increased sampling time until the measurement reached the concentration of the standard after a sample pumping time of 90 s for all of the glucose standard concentration values. Higher pumping speeds and larger diameter sample withdrawing tubes independently reduced the time required to reach the peak glucose concentration measurement; a sampling time of 0.5 min or less could not be measured in any testing setup. The in vivo study showed a good correlation between the glucose concentration measured by the system and the actual standard concentration. The system measurements remained stable throughout a seven-hour continuous testing duration, and the concentrations obtained using the system were identical to those obtained using conventional intermittent blood sampling techniques. 

**Figure 26:**
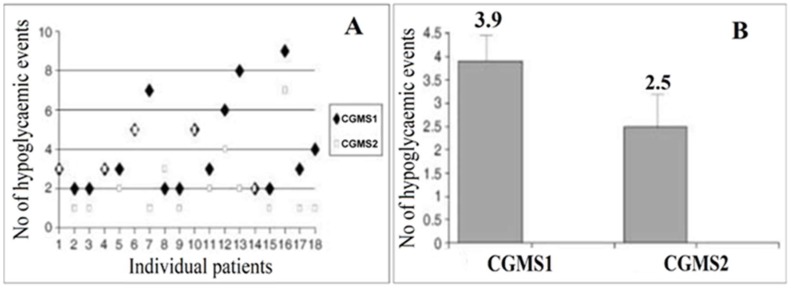
Number of hypoglyceamia events (**A**), (**B**) median number of hypoglyceamic events within 72 h per patient before (CGMS-1) and after (CGMS-2) insulin therapy corrections [92].

Schiaffini et al. evaluated a commercial continuous glucose monitoring system (CGMS) for reduction of hypoglycemia episodes in children with type 1 diabetes mellitus [92]. The MiniMed. Inc. CGMS (Medtronic MiniMed, Inc., Northridge, Los Angeles, CA, USA) was used to monitor glucose levels in twenty-seven diabetic children who obtained standard 4–5 registrations of capillary glycemia per day using the Glucotrend 2 (Roche Diagnostics, Risch-Rotkreuz, Switzerland) glucometer. The average glycemic values measured by CGMS were similar to those obtained using a standard monitoring system (10.58 + 1.92 mmol/L and 10.74 + 1.58 mmol/L). The CGMS system showed a significantly higher number of asymptomatic hypoglycemic events compared to the standard approach (3.6 + 2.3 compared with 0.7 + 0.9) over 72 h, with 26% of patients experiencing hypoglycemic events with a duration less than 30 min, 44% of patients experiencing hypoglycemic events lasting between 30–60 min, and 30% of patients experiencing hypoglycemic events longer than 60 min (Figure 26). The six-week follow-up study indicated that changes in insulin therapy did not significantly affect the incidence of hypoglycemic events; however, a reduction in fructosamine levels was observed. 

Ichimori et al. described a needle-type glucose sensor for real-time monitoring of interstitial and blood glucose concentrations for a wearable artificial endocrine pancreas (WAEP) [93]. The sensor consisted of a polyimide core with a platinum anode in the distal region and a silver cathode in the proximal region, which was encased in a 6% polyurethane and poly(2-methacryloyloxyethyl phosphorylcholine-co-*n*-butyl methacrylate (MPC-co-BMA) membrane. In vitro analysis of the sensor in a 0.9% NaCl solution with varying glucose concentrations (0–500 mg/100 mL) showed a linear relationship between the output current of the sensor and the glucose concentration. A residual current of 2.8 + −1.2 nA was measured in 0.9% NaCl without glucose; a mean output current of 58.0 + 7.2 nA was obtained against a 200 mg/100 mL glucose solution. In vivo studies in diabetes-induced beagles showed a linear relationship (Y = 1.03X + 7.98, r = 0.969) between the interstitial glucose concentration measured by the sensor (Y) and the whole blood glucose concentration measured with a HemCue B-glucose analyzer for 30–350 mg/100 mL (X) (Figure 24). Sensors implanted subcutaneously and intravenously in dogs were used to determine the time delay between interstitial glucose and blood glucose levels. By analyzing the continuous glucose curves for the two anatomical locations, a 6.6 + 1.2 min time delay was determined. 

In order to make continuous glucose monitoring more practical, the use of implantable analyte detection sensors that are coupled to transdermal sensor control units has been proposed [94]. The sensors may be implanted into a vein or artery, subcutaneously, or interstitially; they would be connected to a sensor control unit on the skin of the patient. Detection of glucose levels in blood or interstitial fluid can be monitored through the sensing unit directly or transmitted to another device to provide real-time feedback on blood glucose levels and/or trigger alerts for hypo- or hyperglycemic conditions. Transmitted data can be utilized directly by the patient (e.g., displayed on a telephone, computer, and/or alarm clock) or monitored remotely by caregivers or a watchdog circuit, which triggering controls in a drug delivery system to provide effective and efficient patient care.

## 12. Conclusions

This article summarizes the development of minimally invasive techniques for transdermal drug delivery and sensing of biologically important analytes. Technologies such as electrical stimulation (e.g., reverse iontophoresis and electroporation), micro-fabrication (e.g., microneedles and micro-fluidics), fluorescence-based biosensing, tattoo-based biosensing, and drug delivery have been considered. Iontophoresis has been successfully implemented for enhancing the rate of drug delivery across the skin. The localized drug delivery and minimally invasive biosensing based on microdevices such as microneedles is the recent evolution in the area. The nanomaterial (LaB_6_, gold nanoparticles) loaded robust microneedles offer an effective means for drug administration in the body; the system is modified to be repeatedly switchable, which enables release of a consistent dosage of the drug. Further the scope of ultrasound directed sensing and drug transport has been thoroughly reviewed, the ultrasound based portable devices are a part of routine healthcare services now-a-days. The microfluidic systems like piezoelectric micropumps often serve as efficient route for targeted drug delivery. In addition, the role of optical sensors in detection of various probe molecules and glucose monitoring, by means of light emission and scattering techniques like fluorescence, luminescence and surface enhanced Raman spectroscopies were discussed thoroughly. Overall, various scientific advancements in the area enable the targeted disruption of uppermost layer of skin without harming the sensitive tissues have added the additional appendages with new level of potentials, which renders the transdermal drug delivery with marked influence in medical field.
